# Patterns of Sex Chromosome Differentiation in Spiders: Insights from Comparative Genomic Hybridisation

**DOI:** 10.3390/genes11080849

**Published:** 2020-07-24

**Authors:** Alexandr Sember, Michaela Pappová, Martin Forman, Petr Nguyen, František Marec, Martina Dalíková, Klára Divišová, Marie Doležálková-Kaštánková, Magda Zrzavá, David Sadílek, Barbora Hrubá, Jiří Král

**Affiliations:** 1 Laboratory of Fish Genetics, Institute of Animal Physiology and Genetics, Czech Academy of Sciences, Rumburská 89, 277 21 Liběchov, Czech Republic; dolezalkova@iapg.cas.cz; 2 Laboratory of Arachnid Cytogenetics, Department of Genetics and Microbiology, Faculty of Science, Charles University, Viničná 5, 128 44 Prague, Czech Republic; michaela.pappova.tn@gmail.com (M.P.); formivelkejpan@seznam.cz (M.F.); Dweep2@seznam.cz (K.D.); david.sadilek@natur.cuni.cz (D.S.); barahruba@seznam.cz (B.H.); spider@natur.cuni.cz (J.K.); 3 Department of Molecular Biology and Genetics, Faculty of Science, University of South Bohemia, Branišovská 1760, 370 05 České Budějovice, Czech Republic; petr.nguyen@prf.jcu.cz (P.N.); m.dalikova@gmail.com (M.D.); maggie@prf.jcu.cz (M.Z.); 4 Biology Centre of the Czech Academy of Sciences, Institute of Entomology, Branišovská 31, 370 05 České Budějovice, Czech Republic; marec@entu.cas.cz; 5 Department of Zoology, Faculty of Science, Charles University, Viničná 7, 128 44 Prague, Czech Republic

**Keywords:** achiasmatic pairing, Arthropoda, in situ hybridisation, karyotype evolution, male-specific region, neo-sex chromosome, repetitive DNA, Y chromosome, X_1_X_2_Y, X_1_X_2_0

## Abstract

Spiders are an intriguing model to analyse sex chromosome evolution because of their peculiar multiple X chromosome systems. Y chromosomes were considered rare in this group, arising after neo-sex chromosome formation by X chromosome-autosome rearrangements. However, recent findings suggest that Y chromosomes are more common in spiders than previously thought. Besides neo-sex chromosomes, they are also involved in the ancient X_1_X_2_Y system of haplogyne spiders, whose origin is unknown. Furthermore, spiders seem to exhibit obligatorily one or two pairs of cryptic homomorphic XY chromosomes (further cryptic sex chromosome pairs, CSCPs), which could represent the ancestral spider sex chromosomes. Here, we analyse the molecular differentiation of particular types of spider Y chromosomes in a representative set of ten species by comparative genomic hybridisation (CGH). We found a high Y chromosome differentiation in haplogyne species with X_1_X_2_Y system except for *Loxosceles* spp. CSCP chromosomes exhibited generally low differentiation. Possible mechanisms and factors behind the observed patterns are discussed. The presence of autosomal regions marked predominantly or exclusively with the male or female probe was also recorded. We attribute this pattern to intraspecific variability in the copy number and distribution of certain repetitive DNAs in spider genomes, pointing thus to the limits of CGH in this arachnid group. In addition, we confirmed nonrandom association of chromosomes belonging to particular CSCPs at spermatogonial mitosis and spermatocyte meiosis and their association with multiple Xs throughout meiosis. Taken together, our data suggest diverse evolutionary pathways of molecular differentiation in different types of spider Y chromosomes.

## 1. Introduction

Sex chromosomes represent one of the most intriguing topics of contemporary genetics and evolutionary biology. Investigation of their molecular differentiation is a rapidly evolving research area, continuously bringing new insights and challenges to long-standing evolutionary paradigms [1,2,3,4,5,6,7,8,9,10,11]. According to the generally accepted model, the sex chromosomes usually develop from an autosomal pair after acquisition of a sex-determining gene, which then creates strong linkage with nearby sexually antagonistic genes [12,13,14]. Sex chromosomes are then expected to evolve towards suppression of recombination around the newly established sex-determining region, which allows for the independent evolution of X and Y or Z and W sex chromosome counterparts. The nonrecombining region of the hemizygous sex chromosome (hereafter denoted as allosome, Y or W) usually further differentiates by means of molecular decay of the gene content, accumulation of repetitive DNA, consequent heterochromatinisation, and accompanied morphological changes of this chromosome. In many cases, the allosome shrinks gradually in size, degenerates, and may be potentially even eliminated from the karyotype at some point [13,14,15,16,17].

Among numerous model taxonomic groups suitable for studying the evolution of sex chromosomes, the present study focuses on spiders (Araneae), as their genomes contain very complex and peculiar sex chromosome systems [18,19,20,21,22]. Spiders are an extremely diversified arthropod order with more than 48,000 species [23], which comprises three primary evolutionary lineages—Mesothelae, Mygalomorphae, and Araneomorphae [24,25]. The latter clade is much more diversified than the other two and consists of two lineages known as entelegynes and haplogynes. It is assumed that an ancestral sex chromosome system of spiders is the ♂X_1_X_2_/♀X_1_X_1_X_2_X_2_ constitution (designated also as X_1_X_2_0 system, where 0 means the absence of Y gonosome) [26]. It is also the most common sex chromosome constitution of the most speciose spider clade, entelegyne araneomorphs [20,22]. Some spiders display even more complex, derived multiple X chromosome systems [21], a scenario very unusual in other animal groups [17,27,28]. While multiple X systems of the araneomorphs show up to four differentiated Xs, in mygalomorphs, they exhibit up to 13 X chromosomes [21]. The mechanisms responsible for generating such obscure sex chromosome systems have not yet been explained. Several lines of evidence favour the involvement of sex chromosome nondisjunctions [18,19,29] and fissions, as well as polyploidisation [21].

The presence of Y chromosomes has also been reported in some spiders, though much less frequently. Some of these cases represent neo-sex chromosomes, which evolve by structural chromosome rearrangements between gonosomes and autosomes [18,21,30,31,32,33,34,35]. Furthermore, the Y chromosome is involved in a peculiar X_1_X_2_Y system, which occurs in several families of haplogyne spiders [31,36,37,38]. In all haplogynes described to date, the X_1_X_2_Y system is characterised by a specific structure and unusual achiasmatic pairing during male meiosis. This system is usually formed by two large metacentric X chromosomes and a tiny metacentric Y microchromosome, which together form a trivalent by pairing via ends of their arms, without formation of chiasmata. The phylogenetic distribution of the X_1_X_2_Y system along with a conservative structure of these sex chromosomes suggest its considerable antiquity [31,37]. The origin of this system is unknown. It is hypothesised that the X_1_X_2_0 system of araneomorph spiders arose from the X_1_X_2_Y system by loss of the Y chromosome [39]. In caponiid haplogynes, number of X and Y chromosomes have increased, most probably due to one or two rounds of genome duplication [37].

Remarkably, there is a growing evidence that Y chromosomes are regular elements of spider sex chromosome systems [18,19,21]. In some spiders, the karyotype analysis also revealed a peculiar chromosome pair (hereafter referred to as cryptic sex chromosome pair, CSCP) formed by homomorphic sex chromosomes X and Y that are not morphologically discernible and pair chiasmatically like autosomes, suggesting that they may maintain their homomorphic nature via meiotic recombination [18,19,21]. Analysis of sex chromosome systems in the entelegyne genus *Tegenaria* (Agelenidae) by transmission electron microscopy (TEM) revealed that this pair associates at one end with multiple X chromosomes in male meiosis. This CSCP could in fact represent ancestral proto-XY chromosomes, and the proto-X chromosome could have produced multiple X chromosomes by nondisjunctions [18]. Association of CSCP and X chromosomes is fragile. In consequence, it is often not preserved on preparations of hypotonised cells [18,19]. The follow-up study on the karyotype evolution of mygalomorphs revealed two homomorphic CSCP pairs in some mygalomorphs of the superfamily Avicularioidea. It was hypothesised that the second pair arose by the genome duplication in an ancestor of this clade [21]. In contrast to most other spiders, CSCPs of many mygalomorphs are discernible cytologically due to their specific behaviour, which includes early association of homologs and precocious separation of their chromatids (spermatogonial mitosis), as well as heterochromatinisation (some meiotic phases) [21]. 

Recent progress in spider genomics has led to publication of first spider genome assemblies [40,41] along with the first sequences linked with X chromosomes [42], but so far, no data about the genetic composition of spider Y chromosomes have been acquired.

The present study investigates the differentiation of sex chromosomes in spiders using comparative genomic hybridisation (CGH). CGH has proven to be an excellent and cost-effective method for identifying homomorphic sex chromosomes as well as for gross-scale delimitation of sex-specific chromosomal regions in sex chromosomes of varying evolutionary age [43,44,45,46,47,48,49,50,51,52]. We performed, for the first time, the CGH analysis in spiders, as well as in Chelicerata in general, to determine and compare patterns of molecular differentiation of the Y chromosomes found in homomorphic XY pairs, haplogyne X_1_X_2_Y system, and in neo-sex chromosome systems. Our results shed more light on the sex chromosome evolution in spiders, including support for the presence of homomorphic XY pairs in spider genomes.

## 2. Materials and Methods

### 2.1. Sampling

Individuals belonging to ten spider species (five mygalomorphs and five araneomorphs) were examined (Table 1). Specimens were collected in field or obtained from rearings. Voucher specimens are deposited in the collection of JK (Faculty of Science, Charles University, Prague). Data on locality and developmental stage of analysed male specimens are provided in Table 1. While also at least one female specimen was sampled per each locality (for DNA isolation and CGH probe preparation), only chromosome preparations from male specimens were analysed due to the following reasons: (i) uniformity of male-heterogamety in all spiders analysed to date and (ii) specific behaviour of sex chromosomes in male germline, usually allowing to distinguish sex chromosomes and autosomes.

### 2.2. Chromosome Preparations

Chromosome spreads were prepared from testes of subadult or adult males by plate spreading as described previously [53] except for *Tegenaria ferruginea* (see below). Briefly, tissues were hypotonised in 0.075 M KCl for 20 min (in araneomorphs) or 30 min (in mygalomorphs) and fixed in ethanol–acetic acid or methanol–acetic acid in a 3:1 ratio (*v*/*v*) twice (10 and 20 min; araneomorphs) or three times (6, 10 and 20 min; mygalomorphs). A piece of fixed tissue was then minced in a drop of 60% acetic acid by a pair of tungsten needles and the resulting suspension was spread on a slide placed on a histological plate (40 °C). Preparations of *T. ferruginea* were obtained by a variant of the dropping technique [18]. Slides were stained with 5% Giemsa solution in Sörensen buffer (pH 6.8) for 28 min or left unstained for FISH procedures.

### 2.3. Comparative Genomic Hybridisation (CGH)

#### 2.3.1. Experimental Design and Probe Preparation

Male and female genomic DNAs (gDNAs) were isolated from muscles by (i) phenol/chloroform/isoamylalcohol extraction using PhaseLock Eppendorf tubes (5PRIME, Gaithersburg, MD, USA), (ii) the CTAB method [54] or (iii) Qiagen DNeasy Blood & Tissue Kit (Qiagen, Hilden, Germany).

Male and female gDNAs were differentially labelled by nick translation according to schemes specified in the following section. The optimal fragment size of probes (approx. 200–500 bp) was achieved within the range of 3 to 9 h of incubation at 15 °C. To block the shared repetitive sequences, we used unlabelled competitive DNA prepared from female gDNA of each studied species. Four different sources of female competitive DNA were tested: (i) pure gDNA fragmented on ultrasonic homogeniser, the Sonopuls HD 2070 (Bandelin Electric, Berlin, Germany) (2–20 cycles, 5–10 pulses, 70% power), in order to reach fragments approx. 100–500 bp long; (ii) gDNA amplified with Illustra GenomiPhi V2 DNA Amplification Kit (GE Healthcare, Buckinghamshire, UK) and then fragmented as in the previous case; (iii) blocking DNA enriched by repetitive sequences generated by DOP-PCR following Kubíčková et al. [55]; (iv) C_0_t-1 DNA (i.e., fraction of gDNA enriched with highly and moderately repetitive sequences) isolated by chromatography on hydroxyapatite column [56,57] or according to Zwick et al. [58]. Finally, we prepared a probe cocktail by mixing both male- and female-derived gDNA probes (in vast majority of cases in a 1:1 ratio). To achieve the optimal concentration of the probe in relation to the genome size, we tested 100–1000 ng of both male- and female-derived gDNA probes. Some probe mixtures were prepared without a blocking DNA, but in other cases, female-derived blocking DNA was added in (if not otherwise stated) 10–30-fold excess in relation to the amount of gDNA probes.

#### 2.3.2. CGH

In *Tegenaria ferruginea*, experiments were done essentially following a protocol of Traut et al. [59]. DNA labelling was achieved by nick translation using the Bionick Labelling system (Life Technologies, Karlsruhe, Germany). Hybridisation probes were labelled with either FluorX-dCTP (female DNA) or Cy3-dCTP (male DNA) (both Amersham Life Science, Arlington Heights, IL, USA). The probe cocktail for one slide contained 100 ng of male probe, 100 ng of female probe and 40 µg of unlabelled blocking DNA prepared by sonication of female gDNA as described above. The whole mixture was dissolved in 10 µL of 50% formamide and 10% dextran sulfate in 2× SSC. The slide pretreatment involved incubation in 1% formaldehyde (1 min) to stabilise the chromatin structure. Denaturation of chromosomes was done in 70% formamide in 2× SSC (pH 7) at 72 °C for 3 min. The probe cocktail was denatured at 90 °C (5 min). Hybridisation took place in a moist chamber at 37 °C for three days. Subsequently, nonspecific hybridisation was removed by a stringency wash at 62 °C for 5 min in 0.1× SSC, 1% Triton X-100. Finally, chromosomes were counterstained with 0.2 μg/mL DAPI and mounted in antifade based on DABCO (1,4-diazabicyclo(2.2.2)-octane; Sigma-Aldrich, St. Louis, MO, USA).

Several modifications to the protocol were tested in order to further optimise CGH. As a result, all other species were examined based on a protocol by Symonová et al. [60]. *T*. *ferruginea* has been re-examined using this protocol to confirm previous results. Briefly, slides were aged for 120 min or overnight at 37 °C and then 60 min at 60 °C. Next, RNase A (200 µg/mL in 2× SSC, 60–90 min, 37 °C) (Sigma-Aldrich, St. Louis, MO, USA) and then pepsin (50 µg/mL in 10 mM HCl, 3 min, 37 °C) were applied. These steps were followed by denaturation in 75% formamide in 2× SSC (pH 7.0) (Sigma-Aldrich) at various conditions. For optimisation, we tested temperatures between 68–72 °C, for 2 min 50 sec or 3 min. Male gDNAs were labelled with biotin-16-dUTP (Roche Diagnostics, Mannheim, Germany) or Cy3-dUTP (GE Healthcare), while female-derived gDNAs were labelled with digoxigenin-11-dUTP or Fluorescein-12-dUTP (both Roche) using Nick Translation Mix (Abbott Molecular, IL, USA). For each slide, corresponding amount of competitive DNA (one of the variants listed above) and sonicated salmon sperm DNA (25 μg per 500 ng of each genomic probe; Sigma-Aldrich) were added to male and female genomic probe. DNA from the resulting mixture was precipitated in 96% ethanol, washed in 70% ethanol, air-dried, and redissolved in 20 μL of hybridisation buffer (50% formamide, 10% dextran sulphate, 2× SSC (standard saline buffer), 0.04 M NaPO_4_ (Sodium Phosphate buffer), 0.1% SDS and Denhardt’s reagent). The hybridisation mixture was denatured for 6 min (86 °C) and then either immediately cooled at 4 °C (10 min) or prehybridised at 37 °C (37, 45 or 60 min) to outcompete the repetitive fraction. After application of the probe cocktail on the slide, the hybridisation took place for 72 h (37 °C) in a moist chamber. Post-hybridisation washes were carried out with a high stringency: two times in 50% formamide in 2× SSC (pH 7.0) (42–44 °C, 10 min each) and three times in 1× SSC (42–44 °C, 7 min each). In the case of fluorochrome-labelled probes, slides were subsequently washed in distilled water (1 min) and mounted in antifade containing 1.5 µg/mL DAPI (Cambio, Cambridge, UK). In experiments with hapten-labelled probes, probes were detected either by a combination of Anti-Digoxigenin-FITC [Roche; dilution 1:10 in 0.5% BSA (Bovine Serum Albumin)/PBS)] and Streptavidin-Cy3 [(Invitrogen Life Technologies; dilution 1:100 in 10% NGS (Normal Goat Serum)/PBS)] or by Anti-Digoxigenin-Rhodamin (Roche; dilution 1:15 in 0.5% BSA/PBS) and Streptavidin-FITC [(Invitrogen Life Technologies, San Diego, CA, USA; dilution 1:100 in 10% FBS (Fetal Bovine Serum)/PBS)] respectively, at 37 °C for 60 min. Prior this step, the slides were incubated with 3% BSA (Vector Labs, Burlington, Canada) in 4× SSC in 0,01% Tween 20 (37 °C, 20 min) to avoid the nonspecific binding of antibodies. Finally, chromosomes were counterstained with DAPI as described above. Experiments with altered labelling for male and female gDNAs were included to verify the observed patterns and to exclude any artificial results (influenced, e.g., by the type and dilution of applied antibody). The same or very similar conditions for antibody titration gave already reproducible results in previous studies in vertebrates (e.g., in [49,52]).

### 2.4. Fluorescence In Situ Hybridisation (FISH) with Labelled C_0_t-1 DNA

In order to verify the repetitive nature of the isolated C_0_t-1 DNA fraction, which was used as the most stringent competitive DNA variant, we amplified C_0_t-1 DNA prepared from *Grammostola* using the WGA4 single cell whole genome amplification kit (Sigma-Aldrich). The amplification product was further labelled with biotin-16-dUTP (Roche) or Cy3-dUTP (Jena Bioscience, Jena, Germany) using a WGA3 reamplification kit (Sigma-Aldrich). Both procedures were done according to a modified protocol of Dalíková et al. [9]. The probe mixture for one slide contained 500 ng of labelled C_0_t-1 DNA and 25 μg of sonicated salmon sperm DNA (Sigma-Aldrich). The FISH procedure was performed following a published protocol [61], with a modification for the variant with fluorochrome-labelled probe, as described in the previous section.

### 2.5. Microscopy and Image Analysis

Giemsa-stained slides were observed in an Olympus BX50 microscope. Photographs of the chromosomes were captured under a 100× objective using CCD camera DP71 and software Olympus Cell^D (version 5.1). Software ImageJ (version 1.51u, National Institutes of Health) was used for chromosome counting and measuring. The classification of chromosome morphology was based on the ratio of long and short chromosome arms [62].

FISH and CGH preparations were inspected using either a Provis AX70 Olympus microscope or an Olympus IX81 Cell^R microscope, both equipped with appropriate fluorescence filter sets. Suitable plates were captured under 100× objective. In case of Provis AX70 microscope, images were captured with black-and-white CCD camera (DP30W Olympus) for each fluorescent dye using Olympus Acquisition Software. The digital images were then pseudocoloured (blue for DAPI, red for Rhodamine or Cy3, green for FITC or FluorX) and superimposed with MicroImage software (Olympus, version 4.0). In case of Olympus IX81 microscope, images were photographed using CCD camera Hammatsu ORCA C4742-80-12AG. Pseudocolouration and superimposition were processed by Cell^R software (version Xcellence Rt 2.0.1 Build 6074, Olympus Soft Imaging Solutions GmbH, Olympus Czech Group, Prague, Czech Republic). All CGH images presented here have a unified system of pseudocoloured signals regardless the original experimental scheme—red for male gDNA probe and green for female gDNA probe. Composed images were then optimised and arranged using Adobe Photoshop, versions CS6 and CS7 or ImageJ (version 1.51u; National Institutes of Health, Bethesda, MD, USA).

## 3. Results

### 3.1. Technical Outcomes and General Patterns of CGH

In general, all variants of CGH led to reproducible and similar results in particular species. The optimal settings of CGH were as follows:

1. Hapten-based labelling as it allowed more reliable and reproducible results than direct FITC-dUTP/Cy3-dUTP labelling.
2. 500 ng of each labelled probe per slide, though positive results have been observed also after application of 100 or 300 ng of each genomic probe.
3. Application of (at least) ten times more competitive DNA than each genomic probe, accompanied further by a pre-hybridisation step (37 °C, 37 min). Amplification-derived DNA and especially C_0_t-1 DNA proved to be slightly more efficient than pure female-derived genomic DNA.
4. Denaturation of chromosomes in temperatures not higher than 68 °C in mygalomorphs, while araneomorph chromosomes remained mostly unaffected even after denaturation at 72 °C. Denaturation of mygalomorph chromosomes in temperatures higher than 68 °C often led to damage of chromosomes, chromosome loss and artifacts (especially in meiotic plates).

In each experiment, both male- and female-specific probes showed approximately equal binding to the vast majority of chromosomes in the male complement, with preferential location in the centromeric regions of most chromosomes and in telomeric regions of some elements (yellow signals, i.e., combination of green and red), indicating a high content of repetitive DNA in these regions. Binding of probes to these regions was suppressed to different degrees by protocol modifications, but never fully eliminated. Several other regions were labelled predominantly, but not exclusively, by either male or female probe. Only few locations were found to be stained exclusively by either male or female probe and were placed on certain sex chromosomes or CSCPs, but also, in a few cases, on autosomes.

### 3.2. Differentiation of Mygalomorph Sex Chromosomes

*Dipluridae*

*Linothele megatheloides*

Our results confirm the published data on male karyotype of this species [21], which consists of 86 chromosomes, including six X chromosomes (X_1_X_2_X_3_X_4_X_5_X_6_) and an CSCP. Most chromosomes are biarmed (i.e., metacentric or submetacentric). Metacentric chromosomes of CSCP are the largest elements of the karyotype.

**Figure 1:**
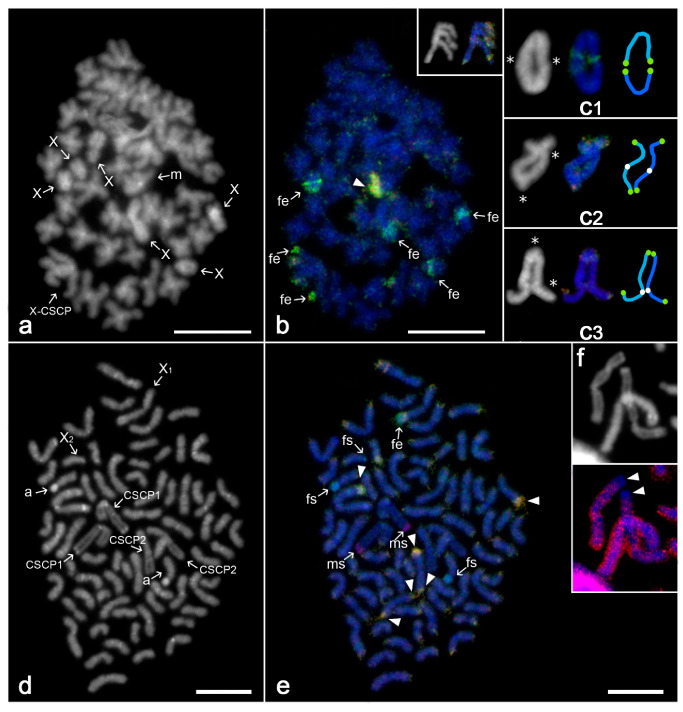
Comparative genomic hybridisation, mygalomorphs *Linothele megatheloides* (**a**)–(**c**) and *Grammostola* aff. *porteri* (**d**)–(**f**), male plates. Abbreviations and symbols: fs—female specific signal (green), fe—female enriched (i.e., biased accumulation of female probe; greenish), ms—male specific signal (red), arrowhead—region highlighted by both male and female probe. (**a**,**b**) *L. megatheloides*. One sister metaphase II including eX chromosome of cryptic sex chromosome pair (CSCP; X-CSCP) and multiple X chromosomes (differing by a tight attachment of chromatids): (**a**) DAPI counterstaining (m—metacentric autosome). Some autosomal centromeres form tiny AT-rich regions; (**b**) comparative genomic hybridisation (CGH). Predominant accumulation of female probe: regions on four X chromosomes, one end of X-CSCP chromosome. Highlighted by both male and female probe: terminal block of a metacentric autosome. Inset: Y chromosome of CSCP, another metaphase II (left DAPI counterstaining, right CGH): the hybridisation pattern resembled the one of X-CSCP; (**c**) *L. megatheloides*, metaphase I, CSCP, pattern of chiasmata [left DAPI counterstaining, asterisk—chiasma; right CGH and schematic drawing of bivalent (one homolog blue and another one light blue, green spots—female-biased signals at the end of chromosomes, white spots—centromeres)]. CSCP includes two chiasmata, which display pericentric (**c3**), intercalar (**c2**) or distal location (**c1**–**3**). (**d**)–(**f**) *Grammostola*, mitotic metaphase (a—chromosomes of an acrocentric pair; CSCP1—chromosome of metacentric CSCP; CSCP2—chromosome of submetacentric CSCP): (**d**) DAPI counterstaining. AT-rich regions: centromeres of some autosomes, centromere and a telomere block of metacentric CSCP; (**e**) CGH. Marked more intensely by female probe (greenish signals): one end of X_1_ chromosome, centromere of putative X_2_ chromosome, centromeres of an acrocentric pair. Highlighted by male probe: chromosomes of metacentric CSCP (ends of the long arms). Highlighted by both male and female probe (yellow signals): bright terminal region at one or both ends of some autosome pairs (most prominent regions marked only); (**f**) hybridisation pattern of female C_0_t-1 DNA probe, chromosomes of metacentric CSCP (CSCP1) and an autosome. DAPI counterstaining (on top) and signal pattern of C_0_t-1 DNA (below). Note the lack of hybridisation at one end (terminal part of long arms) of chromosomes of metacentric CSCP (arrowheads). Bar = 10 µm.

In this species, metaphases of the first and second meiotic division were used for CGH analysis. In metaphase II (Figure 1a), the female probe was enriched in a terminal region on four out of six X chromosomes. One homolog of a large autosomal metacentric pair showed a remarkable yellow terminal region highlighted with both the female and male genomic probes (Figure 1a,b; Supplementary File 1 Figure S1). Chromosomes X (Figure 1b) and Y of CSCP (inset of Figure 1) did not differ by their signal pattern during metaphase II. Both ends of these chromosomes were highlighted predominantly by the female genomic probe (compare Figure 1b and its inset; Supplementary File 1 Figure S1). In metaphase I, chiasmata of CSCP were not confined to a specific region, but randomly distributed along the entire length of chromosomes, similarly to patterns found in autosome pairs. The CSCP bivalent showed usually two chiasmata, with pericentric, intercalar or distal location (Figure 1c).

*Theraphosidae*

*Grammostola aff. porteri*

The male karyotype consisted of 72 chromosomes, including two X chromosomes (X_1_ and X_2_) and two CSCPs, metacentric CSCP1 and submetacentric CSCP2. Karyotype was predominated by biarmed chromosomes. Metacentric X_1_ chromosome belonged to the largest elements of the complement, while acrocentric X_2_ chromosome was identified among the smallest elements (Figure 1d; Supplementary File 1 Figure S1). An acrocentric pair contained a prominent centromeric block. Metacentric X_1_ chromosome involved a subterminal AT-rich block (i.e., region marked intensely by AT-specific fluorochrome DAPI). The centromere and terminal region of the long arm of metacentric CSCP1 were AT-rich too. Homologs of CSCP1 and CSCP2 were located frequently close to each other in spermatogonial mitosis (Figure 1d).

Mitotic plates were used to detect sex-specific regions by CGH. Three chromosome blocks were highlighted by the female probe (green signals), namely, the AT-rich centromere in an acrocentric pair and the centromere of the putative X_2_ chromosome. One female-enriched block not highlighted by DAPI was present on one homolog of a larger submetacentric pair (Figure 1e; Supplementary File 1 Figure S1). On the contrary, the AT-rich terminal block at the long arm of metacentric CSCP1 was labelled by the male probe (red signals) (Figure 1e, Supplementary File 1 Figure S1). Chromosomes of CSCP1 did not differ in the size of this block. Remarkably, this region exhibited a low labelling by the female C_0_t-1 DNA probe (Figure 1f). CGH left a submetacentric CSCP2 without any differential hybridisation pattern (Figure 1e; Supplementary File 1 Figure S1). One or two ends of some autosomes were highlighted by both male and female probes (Figure 1e).

*Poecilotheria formosa*

**Figure 2:**
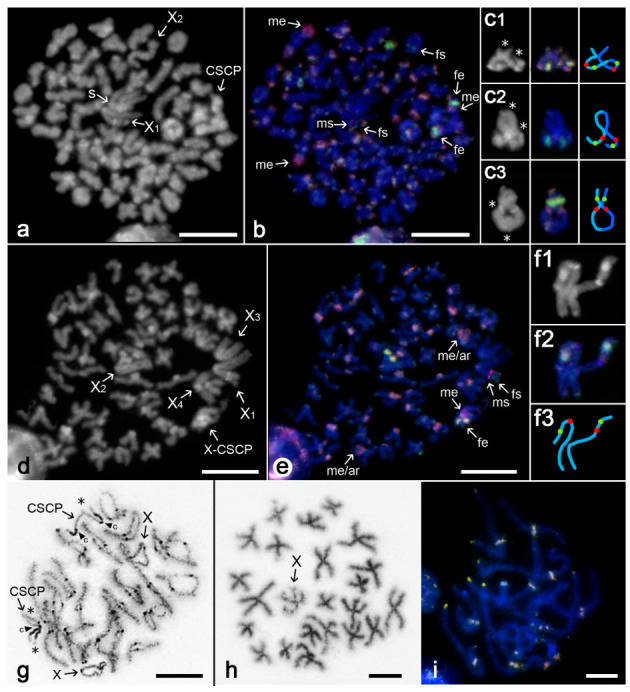
Comparative genomic hybridisation, theraphosid mygalomorphs *Poecilotheria formosa* (**a**)–(**f**) and *Pterinochilus lugardi* (**g**)–(**i**), male plates. Abbreviations: fs—female specific signal (green), fe—female enriched (i.e., biased accumulation of female probe) (greenish), ms—male specific signal (red), me—male enriched (i.e., biased accumulation of male probe) (reddish), s—X chromosome cluster, X-CSCP—X chromosome of CSCP. (**a**)–(**c**) *P. formosa*, metaphase I: (**a**) DAPI counterstaining. Some autosome bivalents exhibit a bright block at one or both ends, X_2_ chromosome is partially released from the X chromosome cluster; (**b**) CGH. Highlighted sex chromosome regions: long arm of X_1_ chromosome (two signals, female and male), CSCP (centromeres stained more intensely by female probe, an intercalar block at short arms highlighted predominantly by male probe). Some autosomes also contain blocks stained more intensely by male probe, and one bivalent has a tiny female-specific signal; (**c**) metaphase I, CSCP, pattern of chiasmata [from left to right: DAPI counterstaining, asterisk—chiasma; CGH; schematic drawing of bivalent (one homolog blue and another one light blue, green spots—female-enriched signals, red spots—male-enriched signals at centromere region)]. CSCP includes two chiasmata with pericentric (**c3**), intercalar (**c1**,**2**) or distal location (**c1–3**); (**d**,**e**) *P. formosa*, metaphase II: X chromosomes (except for X_2_) form a cluster on the plate periphery. Sister chromatids of multiple X chromosomes remain attached: (**d**) DAPI counterstaining. Centromeres of many (usually monoarmed) autosomes are AT-rich. AT-rich sex chromosome regions: X_4_ chromosome (centromeric), X_1_ chromosome (intercalary), CSCP chromosome (intercalary); (**e**) CGH. Highlighted sex chromosome regions: long arm of X_1_ chromosome (one male and one female signal), one arm of X-CSCP (a tiny signal more highlighted by a male probe; other blocks enriched in female probe). Centromeres and telomeres of some autosomes are more highlighted by male probe. In two autosomes, whole short arm is highlighted by this probe (me/ar).; (**f**) *P. formosa*, mitotic metaphase, CSCP chromosomes are aligned in parallel: (**f1**) DAPI counterstaining, (**f2**) CGH, (**f3**) schematic drawing of the association; (**g**)–(**i**) *P. lugardi*: (**g**) pachytene/diplotene transition, two fused plates, Giemsa staining. Centomere regions are formed by a dark knob, arms of each X chromosome associated terminally with each other. CSCP: proximal part of one arm heterochromatic (c—centromere), chiasmata (asterisks) are outside of this region; (**h**) fused sister metaphases II, Giemsa staining. Note biarmed chromosome morphology and undercondensed X chromosome; (**i**) pachytene, CGH, no specific accumulations of genomic probes. Bar = 10 µm.

Our results confirm previously reported karyotype and meiotic sex chromosome behaviour in this species. Karyotype comprises 110 chromosomes including four X chromosomes (X_1_X_2_X_3_X_4_) and CSCP. *P. formosa* has approximately the same portion of biarmed and monoarmed (i.e., subtelocentric or acrocentric) chromosomes. Sex chromosomes are metacentric except for submetacentric X_4_ [19]. Multiple Xs are clustered during male meiosis. While they are usually placed in the middle of the plate during late prophase and metaphase I ([19]; Figure 2a,b), they are on the periphery of the plate at metaphase II ([19]; Figure 2d,e). One X chromosome is often released from the sex chromosome association during meiosis ([19]; Figure 2a–e).

Mitotic and meiotic male plates were used for CGH analysis. During metaphase I, most autosome bivalents displayed equal accumulation of both genomic probes, one bivalent displayed a tiny female-specific signal and few bivalents exhibited slightly male-biased accumulation at one or both ends. A terminal reddish region on two bivalents was of an enormous size. One of these bivalents displayed a size heteromorphism for this region (Figure 2b; Supplementary File 1 Figure S1). This observation is in line with heterochromatin polymorphism reported previously in other male specimens of this species [19]. Two small bivalents displayed a tiny region highlighted exclusively by a female probe. X_1_ chromosome involved two specific tiny intercalar regions, which were placed close to each other. While one of them (AT-rich) was labelled by male probe, another one was marked by female probe. Further, a large terminal segment of X_2_ chromosome was equally marked by both genomic probes; this chromosome was often released from the sex chromosome association (Figure 2a,b; Supplementary File 1 Figure S1). Chromosomes of a single CSCP showed remarkable blocks with equal or slightly female-biased accumulation of genomic probes and a tiny region slightly more enriched for the male probe accumulation (Figure 2a–c; Supplementary File 1 Figure S1).

In metaphase II, centromeres of some autosomes were clearly AT-rich and some sex chromosomes also involved AT-rich regions (tiny intercalar region on the short arm of X_1_ chromosome, centromere of X_4_ chromosome, centromere of CSCP chromosomes). The X chromosome of CSCP was involved in the sex chromosome cluster along with multiple X chromosomes (Figure 2d). The CGH pattern was consistent with the one found in metaphase I but allowed us to more precisely determine the location of specific signals (Figure 2e). The short arm of the X_1_ chromosome contained two specific regions, one highlighted by the male probe and another one by the female probe. The terminal region of the short arm of X_2_ chromosome was marked predominantly by the female probe (Figure 2e; Supplementary File 1 Figure S1).

Analysis of spermatogonial metaphases revealed homologous association of CSCP chromosomes (Figure 2f). During prophase and metaphase I, CSCP exhibited the same pattern of chiasmata as autosome bivalents. The bivalent formed by CSCP contained one or two chiasmata, which showed pericentric, intercalar or distal location (Figure 2c).

*Pterinochilus lugardi*

The male karyotype was formed by 23 biarmed elements including a single X chromosome and CSCP (Figure 2g,h). Sex chromosomes exhibited metacentric morphology. Centromeres were marked by a knob during prophase I in Giemsa-stained preparations. In contrast to previous mygalomorphs, only part of CSCP (proximal region of an arm) was facultatively heterochromatic during transition from pachytene to diplotene. Terminal parts of X chromosome arms were associated with each other during this period (Figure 2g).

Preparations used for CGH contained meiotic plates only. Centromere regions of all pachytene bivalents were highlighted by both male and female probes; they exhibited a bright yellowish fluorescence. One or both ends of most bivalents displayed the same pattern. No male- or female-specific signals could be identified in the chromosome complement (Figure 2i; Supplementary File 1 Figure S1).

### 3.3. Differentiation of Sex Chromosomes in Haplogynes with the X_1_X_2_Y System

*Filistatidae*

*Kukulcania aff. hibernalis*

**Figure 3:**
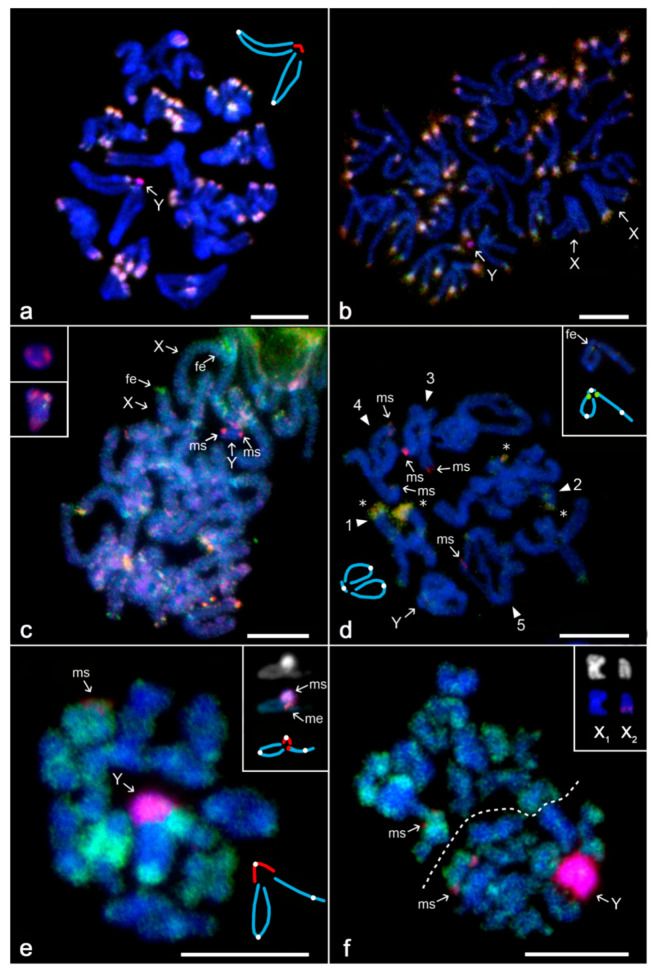
Comparative genomic hybridisation, haplogyne spiders with X_1_X_2_Y system, male plates. Abbreviations: fe—female enriched (i.e., biased accumulation of female probe; greenish), ms—male-specific signal (red), me—male enriched (i.e., biased accumulation of male probe; reddish). Figures **a**,**d**,**e** contain schematic drawings of sex chromosome pairing (white dots—centromeres, red dots—regions highlighted by the male probe). (**a**,**b**) *Kukulcania* aff. *hibernalis*, Y chromosome labelled exclusively by male probe: (**a**) metaphase I; (**b**) fused sister metaphases II. In contrast to autosomes, sister chromatids of X chromosomes are not separated. Most chromosomes exhibit a bright signal at one or both ends (formed by both male and female probe); (**c**) *Loxosceles laeta*, pachytene (Y chromosome with terminal male signals, X chromosomes with a slight prevalence of female signal at one block). Insets: Y chromosomes from other pachytenes—note terminal male signals; (**d**) *L. simillima*, diplotene, Y chromosome without signals. Two chromosome pairs (1, 2) exhibit a prominent terminal bright block (asterisks). Two other pairs (3, 4) have terminal male signal and yet another pair (5) also shows terminal red male signal, which is restricted to one chromatid of one chromosome only (repeatedly observed on multiple nuclei). Inset: X_1_X_2_Y trivalent, one end of right X chromosome is not involved into pairing. Each X chromosome displays a terminal block slightly more accumulated by female probe (greenish signal); (**e**,**f**) *Pholcus phalangioides*: (**e**) metaphase I, Y chromosome marked exclusively by male probe. Inset: sex chromosome trivalent, another metaphase I (*first row* DAPI counterstaining, note a bright fluorescence of Y chromosome; *second row* CGH; Y chromosome is marked exclusively by male probe, while region on X_2_ chromosome involved into pairing exhibits a slight preferencefor hybridization of male probe; *third row* schematic drawing); (**f**) two sister metaphases II (separated by a dashed line), Y chromosome and a terminal region of an autosome pair highlighted exclusively by male probe. Inset: X chromosomes from another metaphase II, DAPI counterstaining (*first row*) and CGH (*second row*, note a terminal signal at long arm of X_2_ chromosome, stained more by a male probe). Bar = 10 µm.

The male karyotype contained 11 pairs (Figure 3a) of biarmed chromosomes (including both autosomes and potential but undetected CSCP) (Figure 3b) and sex chromosomes X_1_, X_2_, and Y. While X chromosomes are large metacentrics of similar size, the Y chromosome is a tiny element whose morphology could not be resolved (Figure 3b). X chromosomes paired achiasmatically by ends of both arms with the Y chromosome during male prophase and metaphase I (Figure 3a). X chromosomes remained associated during metaphase II. Chromatids of each X chromosome remained attached during this period (Figure 3b). Most chromosomes contained AT-rich block at one or both ends. Moreover, Y chromosome and centromeres of several other chromosomes were also AT-rich, including one X chromosome (Supplementary File 1 Figure S1). With CGH, chromosomes bore yellowish regions (highlighted by both male and female probe) at one or both ends, except for one X chromosome and Y chromosome (Figure 3b). The Y chromosome was marked exclusively by the male probe (Figure 3a,b).

*Sicariidae*

*Loxosceles laeta*

The male set consisted of 23 biarmed chromosomes, including the X_1_X_2_Y sex chromosome system (Supplementary File 2 Figure S2c,d). While X chromosomes were metacentric, Y showed submetacentric morphology (Supplementary File 2 Figure S2c,d). Sex chromosomes paired by their ends without chiasmata during male meiosis (Supplementary File 2 Figure S2d). Preparations used for CGH experiments contained plates of the prophase I only. At pachytene, one end of some bivalents was highlighted by both the male and female probes as bright yellowish blocks. Some other chromosomes, including putative Xs, exhibited a telomeric block enriched for the accumulation of the female probe. During this period, the male genomic probe marked terminal parts of Y chromosome (Figure 3c).

*Loxosceles simillima*

Obtained information on karyotype and meiosis is consistent with published data [31]. The male karyotype consists of 19 biarmed chromosomes. The sex chromosomes include two large metacentric X chromosomes and a tiny metacentric Y chromosome, which pair achiasmatically by both ends. Pairing of one X chromosome is often disrupted at one end (Figure 3d).

Meiotic plates were used to determine the pattern of sex-specific signals by CGH. The large region at one end of two chromosome pairs was yellow due to excessive binding of both probes to this area. The Y chromosome was not marked by the male probe. However, male-specific signals were observed at terminal regions of several chromosome pairs. Analysed individuals differed in their patterns of these signals. In one specimen, chromosome pairs bearing a large yellow terminal region were without male signal (Figure 3d). In another spider, one of these pairs contained terminal male signals. Furthermore, one chromosome pair was heterozygous for male signals (not shown). In some cases, one of two sister chromatids showed reduced or no signal (Figure 3d).

*Pholcidae*

*Pholcus phalangioides*

Our data on karyotype and meiotic sex chromosome behaviour are consistent with published information [31]. Males exhibit 25 biarmed chromosomes. Sex chromosomes comprise three large elements: metacentric X_1_, submetacentric X_2_, and metacentric Y. In contrast to previous X_1_X_2_Y species, only one X_2_ arm is involved in the meiotic pairing.

Preparations containing meiotic plates were used for CGH (Figure 3e,f; Supplementary File 1 Figure S1). The Y chromosome was strongly painted by the male probe, as shown at metaphase I (Figure 3e) and metaphase II (Figure 3f). The X_2_ sex chromosome contained a block slightly enriched for the accumulation of male probe; this block participated in the sex chromosome pairing in the trivalent (inset of Figure 3e). Moreover, one end of another chromosome pair was marked predominantly by the male probe (Figure 3f).

### 3.4. Differentiation of Neo-Sex Chromosomes

*Tegenaria ferruginea (Araneomorphae, Entelegynae, Agelenidae)*

**Figure 4:**
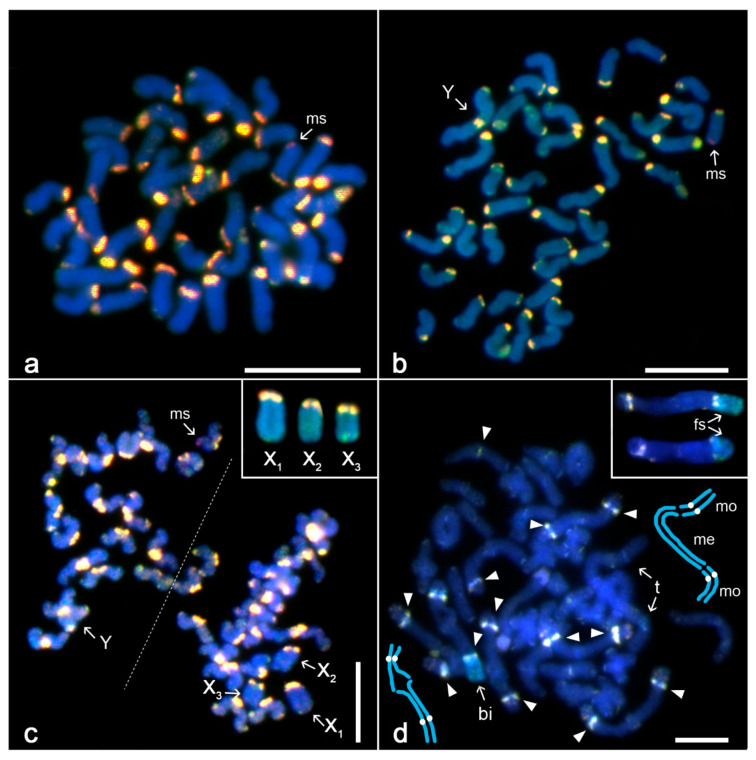
Comparative genomic hybridisation, spiders with neo-sex chromosome systems, male plates. Abbreviations: ms—male signal (red), fs—female signal (green). (**a**)–(**c**) *Tegenaria ferruginea*, centromeres are highlighted by both probes as a yellow block. In some chromosomes an arm is also terminated by yellow block. A small acrocentric chromosome exhibits male terminal signal on the long arm: (**a**,**b**) mitotic metaphase. Y chromosome bears two yellow blocks, centromeric and telomeric; (**c**) two sister metaphases II (separated by a dashed line). In contrast to other chromosomes, sister chromatids of multiple X chromosomes are attached. Inset: multiple X chromosomes, another metaphase II. (**d**) *Atrophothele socotrana*, diplotene. Centromeres of some bivalents are expressed as yellowish blocks (most prominent blocks are marked by arrowhead). Note a slightly heteromorphic bivalent (bi) and a trivalent (t). At left: schematic drawing of the heteromorphic bivalent (white dots—centromeres). At right: schematic drawing of the trivalent (me—metacentric chromosome, mo—monoarmed chromosome, white dots—centromeres of monoarmed chromosomes). Inset: two heteromorphic bivalents from other diplotene plates. Right chromosome of bivalent contains terminal (upper bivalent) or pericentric (lower bivalent) green female signal. Bar = 10 µm.

Our data are consistent with previously reported karyotype (2n♂ = 40, X_1_X_2_X_3_X_4_X_5_Y, with acrocentric chromosomes except for metacentric Y; [18], Figure 4a,b).

The preparations used for CGH contained both spermatogonial mitoses and meiotic plates (Figure 4a–c, Supplementary File 1 Figure S1). With CGH, centromeres and some telomeres of mitotic and meiotic chromosomes were expressed as bright yellow regions, which reflects an excessive binding of male and female probes (Figure 4a–c). The distal end of X_3_ chromosome showed a slight prevalence of female probe accumulation (Figure 4c). Y chromosome was without male-specific signals (Figure 4b,c). The centromere and one end of the Y chromosome were labelled with both probes, which formed yellow blocks of hybridisation signals. Remarkably, the distal end of one chromosome of a short acrocentric pair was highlighted by the male probe (Figure 4a–c; Supplementary File 1 Figure S1).

*Atrophothele socotrana (Mygalomorphae, Barychelidae)*

The male karyotype comprised 68 chromosomes. Although karyotype was predominated by monoarmed chromosomes, the majority of large chromosomes was biarmed (Supplementary File 3 Figure S3). Mitotic and meiotic plates did not contain heterochromatic chromosomes (Supplementary File 2 Figure S2e,f). Late prophases I comprised 34 elements. Most elements were bivalents. One bivalent consisting of biarmed chromosomes was slightly heteromorphic. The short arm of one chromosome was apparently longer than the short arm of its homolog (Supplementary File 2 Figure S2f). Besides bivalents, prophases I also included a chain-like trivalent, which was composed of a central metacentric element flanked by two monoarmed chromosomes (Supplementary File 2 Figure S2f). Given the 2n = 68 and the presence of 34 elements in prophase I, where one element is the trivalent, another element must therefore be a univalent (not recognisable unambiguously from bivalents). Segregation pattern of chromosomes during the first meiotic division (one product with 33 and another with 35 chromosomes; Supplementary File 3 Figure S3) suggests that trivalent and univalent are, in fact, four sex chromosomes where metacentric chromosome of the trivalent is Y element; chromosomes flanking Y chromosome and univalent are X elements. While the meiotic product with 33 chromosomes contains the Y chromosome, the product with 35 chromosomes includes three X chromosomes.

Preparations used for CGH contained prophases I only. With CGH, centromeres of some chromosome pairs were labelled by both male and female probes. The female probe highlighted a region of the heteromorphic pair, specifically the short arm of a single chromosome (Figure 4d; Supplementary File 1 Figure S1). Other chromosomes of this species were not labelled with the male or female probe.

## 4. Discussion

**Figure 5:**
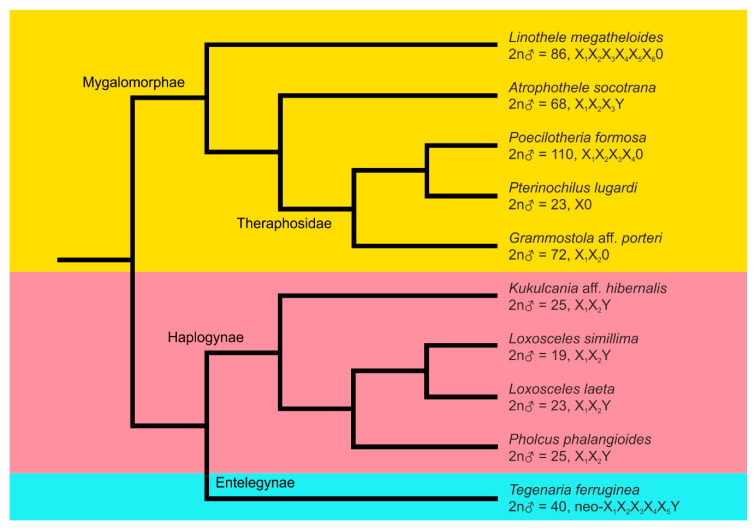
Phylogenetic relationships and karyotype characteristics of inspected spider species. The simplified phylogenetic tree was constructed following Wheeler et al. [63] except for Theraphosidae [64]. See Fernandez et al. [65] for proposed times of divergence of major lineages. Lineages: Mygalomorphae (orange background), Haplogynae (pink background), Entelegynae (light blue background).

Spiders are an excellent invertebrate group for studying sex determination [40]. They are renowned for their peculiar and very complex sex chromosome determination, including several different X chromosomes [18,19,20,21,22]. As suggested by the current studies, spider sex chromosome systems are even more complex than previously anticipated, as they most likely contain one or two homomorphic XY pairs [18,19,21]. Besides this, Y chromosome is involved in a peculiar X_1_X_2_Y system of haplogyne spiders and in the formation of neo-sex chromosomes arising by rearrangements between sex chromosomes and autosomes. Using CGH, we have characterised the degree of divergence of the sex chromosomes in selected spiders with Y chromosomes involved in different sex chromosome systems. Our study represents the first endeavour to analyse spider sex chromosomes by means of this molecular cytogenetic method. The phylogenetic relationships among the studied spider species, along with the information about their 2n and sex chromosome systems, is given in Figure 5. In five out of ten spider species under study, karyotypes and sex chromosomes were described for the first time (Table 1). Similar to almost all other spiders [21,22], male meiosis of the species under study is chiasmatic. Achiasmatic pairing concerns only canonical sex chromosomes (X_1_X_2_Y system of haplogyne spiders and multiple X chromosome systems).

### 4.1. Signal Patterns on Autosomes

In theory, by CGH we might reveal, mainly or exclusively, differences in repetitive DNA accumulation between sex chromosomes [59]. Nonetheless, our data show that sex-specific or sex biased signals were also detected on autosomes of some species. In haplogynes, a specific case of interindividual variability in *Loxosceles* *simillima* is discussed below. In a single entelegyne under study, *Tegenaria ferruginea*, one male-specific signal is present on a single autosome. In mygalomorphs, the centromeric blocks of one (*Linothele*) or several autosome pairs (*Grammostola*) were marked predominantly by a female probe, which may reflect the expansion of X chromosome-enriched class of repetitive DNA to the centromeres of some autosome pairs by ectopic (i.e., nonallelic) recombination or an occurrence of centromere repetitions, whose sequence motif is very similar to those located on the X chromosome. A similar mechanism could be responsible for the predominance of male-specific probe hybridisation in the terminal regions of two autosomal pairs of *Poecilotheria*. The location of these regions is consistent with the previously reported C-banding pattern [19], which indicates their position within constitutive heterochromatin. These patterns may be explained by intraspecific variability in abundance of particular repetitive DNA classes in the genomes of studied spiders. It has been repeatedly shown that the amount and distribution of heterochromatin may highly differ among individuals of the same species within a single population—both on autosomes (such as in a geometrid moth *Abraxas grossulariata* [51]) and sex chromosomes (e.g., in necrophagous fly *Lucilia sericata* [66]). Such loci usually contain highly variable classes of tandem repeats, especially satellite DNA and microsatellites, which are prone to fast sequence evolution (particularly satellite DNA) and rapid copy number variation [67,68]. For instance, in maize, it has been shown that microsatellite megatracts can differ not only among lines, but even among siblings [69]. Hence, we show the possible limits of the CGH-based analysis in spiders and stress the necessity of (i) proper locality-specific sampling and (ii) former identification of spider sex chromosomes by other methods as de novo identification of cryptic sex chromosomes by CGH might be largely hampered by hybridisation “noise” on autosomes. Still, in cases of well-recognised sex chromosomes and CSCPs, the analysis can be narrowed to these elements, as we do below.

### 4.2. Evolution of Sex Chromosomes in Mygalomorphs

Sex chromosome systems in members of the mygalomorph superfamily Avicularioidea are very complex in their composition. Besides one or several X chromosomes, karyotypes of mygalomorphs contain one or even two homomorphic CSCPs, which in some mygalomorph clades exhibit the specific behaviour in the male germline [21]. Our data support the hypothesis that chromosomes forming CSCP exhibit homologous parallel pairing already at spermatogonial mitosis [21]. Furthermore, our data suggest that multiple X chromosomes and CSCP chromosomes may be associated not only during the first, but also in the second meiotic division (Figure 1a, Figure 2a, Figure 3b and Figure 4c).

Sex chromosomes of mygalomorphs studied herein by CGH showed a diversified pattern of sex chromosome-specific signals. Intriguingly, apart from *Pterinochilus* where no biased hybridisation patterns were observed (Figure 2i), X chromosome univalents of mygalomorph males often contain regions highlighted more intensely by female-derived probe (Figure 1b,e). We suppose that these regions are enriched in repetitive DNA and biased hybridisation of female-derived probe on X chromosomes might be explained by a copy number difference between males and females in this repetitive fraction. Spider males possess just one homolog of the X chromosome, whilst females have two X copies. As a result, the female probe may be enriched in X-chromosome linked repetitive DNA compared to male probe. Zrzavá et al. [51] observed a similar hybridisation pattern on the paired sex chromosome Z of geometrid moths. However, unlike spiders, these insects exhibit double dose of Z chromosome in males compared to females.

Our results further show an unusual pattern of sex-specific regions on the X_1_ chromosome of *Poecilotheria* (Figure 2b,e). Besides an area highlighted exclusively by female probe, a long arm of the X_1_ chromosome contains also a tiny male-specific signal. It may be speculated that this region could have been translocated to the X_1_ chromosome from the CSCP by means of ectopic recombination or transposition. These mechanisms are known to give rise to similar transfers between sex chromosomes and autosomes in various animal groups [6,70,71,72]. Alternatively, since CGH provided different patterns of hybridisation signals between male and female probe even on autosomes, this male-specific signal on the X_1_ chromosome might reflect the intraspecific variability in expansion of repeats which might not mirror sex-linked differences. In humans, for instance, it has been shown that each individual has about 76–85 de novo mutations in short tandem repeats with 2–4 bp motifs [73].

Mygalomorph CSCPs usually exhibited a specific hybridisation pattern as they contained sex specific signals generally more often than autosomes; however, there was no apparent difference between X and Y in this respect. Some regions were marked exclusively by the male probe (one end of one CSCP of *Grammostola*; Figure 1e), while other ones displayed stronger hybridisation of the female probe (both ends of *Linothele* CSCP; Figure 1b). Finally, CSCP of *Poecilotheria* carries one tiny region with male-biased accumulation and a remarkable block where both probes accumulate, but the hybridisation of the female probe is slightly stronger (Figure 2b–d,f). Centromeric and telomeric location of these signals indicates their heterochromatic nature and predomination of repetitive sequences. Regions marked preferentially by the male probe are AT-rich. Lack of differential hybridisation between CSCP counterparts, identical morphology of these chromosomes and distribution of chiasmata, which can be formed along the entire or almost entire chromosome length—these observed patterns together suggest a very low differentiation level between X and Y chromosomes, with an early stage of divergence of associated repetitive DNA. In some cases, the corresponding region of the X and Y chromosome is enriched in female probe, probably due to similar reasons as hypothesised above (i.e., double dose of X-linked repetitive DNA in female probe compared to male probe). In other cases, corresponding regions on X and Y chromosomes are both marked predominantly by the male probe (*Grammostola*; Figure 1e). In this case, preferential binding of the male probe could be, by analogy, caused by enrichment of certain repetitive DNA in the male genome, which may be either (i) sex-linked or (ii) resulting from intraspecific variability. The former possibility seems more likely as the signal is restricted to CSCPs.

Notably, one or both CSCPs of many avicularioid mygalomorphs are completely facultatively heterochromatic during early prophase of the first meiotic division ([21] and Figure 2g). There is growing evidence that meiotic heterochromatinisation prevents ectopic recombination between structurally differentiated sex chromosomes [74,75,76]. However, this seems not to be applicable to CSCPs as these chromosomes exhibit very low differentiation, and their recombination pattern seems not to differ from standard autosome pairs. Considering the supposed duplication of CSCP in the ancestral members of avicularioid mygalomorphs [21], heterochromatinisation of CSCP could prevent recombination between chromosomes belonging to different CSCP pairs. According to another hypothesis, multiple X chromosomes of spiders are produced by nondisjunctions of X chromosome of CSCP, and newly emerged sex chromosomes subsequently undergo gradual structural differentiation [18]. If so, heterochromatinisation of CSCP could also impede recombination between CSCP and multiple X chromosomes. These hypotheses are in line with a standard recombination pattern of CSCPs.

### 4.3. Evolution of the X_1_X_2_Y System in Haplogyne Spiders

The peculiar X_1_X_2_Y system is one of most frequent sex chromosome determinations in haplogyne spiders. Conservative morphology and specific achiasmatic pairing of chromosomes X_1_, X_2_, and Y strongly suggest that spider taxa with the X_1_X_2_Y system form a monophyletic clade [31]. Distribution of the X_1_X_2_Y system based on a recent phylogenomic tree [77] suggests that this sex chromosome determination is probably ancestral for haplogynes. It could arise even earlier in spider evolution, namely, in ancestral araneomorph spiders [39]. It was suggested formerly that the X_1_X_2_Y system evolved via rearrangements between autosomes and sex chromosomes [78]. If so, it would represent an ancient neo-sex chromosome system. The ancestral form of the haplogyne X_1_X_2_Y system was probably formed by two metacentric X chromosomes of similar size and a metacentric Y microchromosome, which exhibited a specific pairing by ends of both arms [31]. This pattern of the X_1_X_2_Y system is still retained in many lineages of haplogynes. In accordance with a long evolutionary history of the Y microchromosome, this element exhibits a high degree of differentiation in *Kukulcania hibernalis*, as it is labelled exclusively by the male-specific probe (Figure 3a,b). Despite a considerable increase of Y chromosome size in *Pholcus phalangioides*, this element retains the pattern of differentiation found in *Kukulcania* (Figure 3e,f). The Y chromosome of *P. phalangioides* is reported to be formed exclusively by constitutive heterochromatin [31]. Therefore, an enlargement of the Y chromosome in this spider could be ascribed to considerable accumulation of repetitive sequences, which usually predominate in constitutive heterochromatin [67,79,80]. Cases of enlargement of sex-limited sex chromosomes due to repetitive DNA accumulation are known in several organisms [8,45,81,82,83,84,85,86,87,88]. As described in diverse animal taxa, these sequences could even expand from the Y chromosome via ectopic recombination and/or transposition [6,70,71,72]. In *Pholcus*, such a scenario might be indicated by the slightly more intense accumulation of male probe at the end of X_2_ chromosome; this region is involved in pairing of X_2_ and Y chromosomes. Such a localisation suggests a possible role of these sequences in achiasmatic pairing of sex chromosomes in meiosis, as described for heterochromatic regions in diverse animals [89].

Remarkably, Y microchromosome of another haplogyne with a X_1_X_2_Y system, *Loxosceles laeta*, exhibits male-specific sequences only at the terminal regions (Figure 3c) and a congener *L. simillima*, even lacks any male-specific signal on its Y chromosome (Figure 3d). These results point to interspecific variability in the trajectory of molecular degeneration, the structure and composition of Y chromosomes in these taxa, similar to what has been documented in closely related species of butterflies [44,90,91]. The absence of male-specific signal on the Y chromosome of *L. simillima* may reflect the absence of a dominant class of repetitive DNA on the Y chromosome of this spider. Although the absence of CGH sex-specific signal is usually detected on low-differentiated, homomorphic sex chromosomes [92,93,94], it was also observed on differentiated, heteromorphic sex chromosomes of some animals [45,47,50]. For instance, in oplurid reptiles [47] the minute Y chromosome was found to harbour male-linked genes after quantitative PCR, while the repetitive DNA that usually accounts for CGH resolution [95,96] was eliminated. Furthermore, the lack of positive male-biased hybridisation in *L. similima* may reflect additions of autosomal material to sex chromosome as repeatedly reported in diverse animals [97,98,99]. Such a process might enlarge regions of pairing between sex chromosomes [100]; however, in the case of *L. simillima*, the original mode of sex chromosome pairing is not affected. Addition of autosomal material to a heterogametic sex chromosome could also lead to dedifferentiation of this element, which might facilitate its revitalisation and a purge of accumulated heterochromatin [3,101]. However, unlike the mentioned examples from lower vertebrates, the mechanism through which the rejuvenation process may operate in invertebrates is unknown.

The pattern of Y chromosome differentiation in *L. laeta* might represent an intermediate step between patterns found in *L. simillima* and the other haplogynes. Restriction of male-limited regions to telomeres in *L. laeta* is intriguing. In some butterfly species, female-limited regions are confined to the internal part of the W sex chromosome due to homogenising ectopic recombinations acting preferentially on (sub-) telomeres [102,103]. The pattern found in *L. laeta*, which is opposite to that found in butterflies, may reflect action of other mechanisms than ectopic recombinations. It could be, for example, a consequence of replacement of the central part of the Y chromosome by a region from the autosome or CSCP by chromosome rearrangement.

Although we did not detect male-specific signals on the sex chromosomes of *L. simillima*, these signals were revealed on several other chromosome pairs of this spider. Given the terminal location of these signals and interindividual variability, the most probable explanation would be the variability in repetitive DNA content between individuals [67,68,69,79], with no link to sex chromosome differentiation. In one individual, the male signal of one chromosome was restricted to one chromatid, which indicates an involvement of sister chromatid exchanges (SCE) in generating the size polymorphism of heterochromatin blocks and therefore considerable heterochromatin dynamics in this species (for details concerning the mechanism of SCE, see [104]). Nonetheless, we cannot entirely exclude the possibility that certain chromosome pair of *L. simillima* bearing male-specific signals might in fact represent CSCP as we could not distinguish this pair from the rest of the chromosome complement. This scenario might also apply to *Pholcus*, which also exhibits additional male-specific signals outside the X_1_X_2_Y system. In contrast to *Loxosceles*, these signals are restricted to a single chromosome pair. This phenomenon warrants further investigation with finer-scale molecular methods.

The last question linked to the observed results in haplogynes reads: why does the Y chromosome only persist in some haplogyne clades? The function of the Y chromosome and the evolutionary forces that underpin its persistence in some haplogyne lineages are unknown. It might be hypothesised that a tiny Y somewhat promotes the stability of sex chromosome trivalent during meiosis. However, this idea is contradicted by the repeated origin of the X_1_X_2_0 system by the loss of the Y chromosome during the evolution of haplogynes, whereas X chromosomes retain the original mode of achiasmatic pairing [105]. It is tempting to hypothesise that the persistence of the Y chromosome in some haplogyne clades could be supported by rejuvenation of this element by material derived from other autosomes or CSCP, which would increase the selection potential of Y chromosome and slow down its degeneration. To achieve a deeper understanding of the Y chromosome’s function and evolution in haplogynes, it will be crucial to analyse the genetic content persisting on this element through finer scale cytogenomic approaches and to compare the evolutionary dynamics and stability of spider Y sex chromosome in chiasmatic and achiasmatic systems, similarly to what has been done in beetles [100].

### 4.4. Evolution of Neo-Sex Chromosome Systems in Spiders

Formation of neo-sex chromosomes seems to be a relatively rare event in the evolution of spiders [20,22], being currently reported in about 40 out of 868 karyotyped spider species [106]. These chromosomes have been described in representatives of both primary clades of opisthothele spiders, i.e., mygalomorphs [21,32] and araneomorphs [18,30,31,33,34]. Evolution of neo-sex chromosomes in spiders might be very complex. These chromosomes can arise by rearrangements involving not only Xs and autosomes, but also CSCP(s) [18]. Taking into account the difficult detection of CSCP(s), neo-sex chromosomes of some spiders could represent in fact the products of rearrangements between Xs and CSCP(s).

The evolution of neo-sex chromosomes usually follows the general principles of sex chromosome differentiation, including cessation of recombination between both counterparts and subsequent erosion of genetic content and overall degeneration of the allosome. Therefore, we applied the CGH-based analysis to assess the differentiation of Y chromosome, which is involved in the neo-sex chromosome system X_1_X_2_X_3_X_4_X_5_Y of a European agelenid, *Tegenaria ferruginea*. Sex chromosomes of this spider arose from the system formed by three X chromosomes and an CSCP via Robertsonian translocation between the Y chromosome of CSCP and an autosome. Although the X_1_ X_2_X_3_X_4_X_5_Y system was found in all populations of *T. ferruginea* studied so far, encompassing the localities from Germany, Czech Republic, Slovakia ([18], this study), and Greece (this study), it was not revealed in closely related species. This evolutionary pattern suggests a relatively recent formation of the X_1_X_2_X_3_X_4_X_5_Y system, which is probably an apomorphy of *T. ferruginea*. In some organisms, differentiation of neo-Y or neo-W chromosomes starts early after their origin [82,107,108,109,110,111]. Despite this, our data did not reveal any differentiation of Y chromosome of this species by the male-specific probe, which resembles the pattern found in neo-sex chromosomes in some other CGH-based studies [50,112,113]. Instead, the male probe differentiated a distal end of an acrocentric chromosome, which is involved in the formation of standard acrocentric bivalent during meiosis and the most likely explanation for this observation is the intraspecific variability in repetitive DNA content as discussed above in Section 4.1.

Our data also suggest a complex sex chromosome system in the barychelid mygalomorph *Atrophothele*. Comparison of male mitotic and meiotic data suggests a presence of three Xs and one Y chromosome in the karyotype of this spider. During the prophase and metaphase I, two X chromosomes and a single Y chromosome form trivalent, while the remaining X chromosome stands apart as univalent. Hypotonisation of meiotic cells probably artificially led to the collapse of achiasmatic pairing of the sex-trivalent and univalent. The presence of a heteromorphic bivalent indicates that the sex chromosome system of this spider could be even more complex. To decipher the structure of sex chromosome system in *Atrophothele*, we analysed its chromosome plates by CGH. The female-derived probe partially differentiated one chromosome of the heteromorphic bivalent, which could reflect X chromosome nature of this element. If so, the heteromorphic bivalent could represent CSCP. Origin of the sex chromosome system of *Atrophothele* is unclear. The ancestral male karyotype of the mygalomorph superfamily Avicularioidea has probably contained four multiple X chromosomes, which form univalents during meiosis [21]. The single X chromosome univalent of *Atrophothele* could therefore represent the original X chromosome, which did not take part in rearrangements between X chromosomes and other chromosomes. The sex chromosome trivalent could arise by rearrangements among remaining X chromosome(s) and other chromosomes. This process could also include CSCP. To determine the origin and composition of the sex chromosome system in *Atrophothele*, an ultrastructural study on sex chromosome pairing during meiosis of heterogametic sex is needed. In contrast to the standard preparation of chromosomes, this approach is more suitable to preserve fragile associations of CSCP and the other sex chromosomes. Another source of information to elucidate the origin of sex chromosomes in *Atrophothele* could be the analysis of related mygalomorphs. Remarkably, another African barychelid, *Cyphonisia*, exhibits a neo-XY system, which also represents a very derived system in mygalomorph spiders [21]. Therefore, the barychelid lineage possessing derived sex chromosome systems could be quite diversified and promising for further investigation at a greater taxonomic scale.

## 5. Conclusions

The goal of the present study was to analyse the molecular differentiation of particular types of spider Y chromosomes by comparative genomic hybridisation. We studied differentiation of CSCPs (four mygalomorph spiders whose CSCPs are detectable cytologically), the X_1_X_2_Y system (four haplogyne spiders), and neo-sex chromosome systems (one mygalomorph and one entelegyne representative). The major outcome is that the CGH method may provide reliable data about spider sex chromosome differentiation, but only in a limited set of spider species. More specifically, a high degree of Y chromosome differentiation was found in haplogyne spiders with the X_1_X_2_Y system (*Pholcus phalangioides*, *Kukulcania* aff. *hibernalis*), which is in line with the absence of recombination between X and Y chromosomes and with presumed considerable age of this system. Surprisingly, there are also haplogyne species with a low (*Loxosceles laeta*) or even no Y chromosome differentiation (*L. simillima*), at least based on the CGH patterns. Furthermore, our study revealed a low Y chromosome differentiation in analysed neo-sex chromosome systems, which may reflect their short evolutionary history. Our observations support the notion of nonrandom alignment/association and homologous pairing of CSCP chromosomes at spermatogonial mitosis and at meiosis, as well as their specific behaviour in the male germline. Although CSCP chromosomes often include regions highlighted more by one sex-specific probe, X and Y chromosomes do not differ by pattern of these signals, which suggests a low differentiation of these chromosomes. Lastly, positive signals unrelated to sex were often recorded, stressing the need for accurate identification of sex chromosomes prior to interpretation of the obtained signal patterns. Autosomal signals could be assigned to intraspecific variability in the number of specific classes of repetitive DNA and their dynamics in spider genomes. Taken together, our data suggest a specific evolutionary differentiation of different types of spider Y chromosomes, which warrants further investigation by finer-scale molecular methods.

## Figures and Tables

**Table 1:** List of analysed spider species.

Species	Primary Clade: Family	Number and Stage of Male Specimens ^a^	Locality or Source	2n ♂	Male Sex Chromosome System	Reference
*Atrophothele socotrana*	Mygalomorphae: Barychelidae	1 AD (1 AD)	Yemen, Socotra Isl., Firmihin plateau	68	X_1_X_2_X_3_Y + CSCP?	this study
*Linothele megatheloides*	Mygalomorphae: Dipluridae	1 SAD, 1 AD	breeding	86	X_1_X_2_X_3_X_4_X_5_X_6_ + CSCP	[21]
*Grammostola* aff. *porteri*	Mygalomorphae: Theraphosidae	1 AD, 1 SAD (5 AD)	Chile, Limarí province, Coquimbo area, Punitaqui	72	X_1_X_2_ + 2 CSCP	this study
*Poecilotheria formosa*	Mygalomorphae: Theraphosidae	1 AD	breeding	110	X_1_X_2_X_3_X_4_ + CSCP	[19]
*Pterinochilus lugardi*	Mygalomorphae: Theraphosidae	1 AD, 1 SAD (2 AD)	breeding	23	X + CSCP	this study
*Kukulcania* aff. *hibernalis*	Araneomorphae, Haplogynae: Filistatidae	2 AD	breeding	25	X_1_X_2_Y	this study
*Loxosceles simillima*	Araneomorphae, Haplogynae: Sicariidae	2 AD, 1 SAD	Republic of South Africa, Free State, Ndumo Game Reserve	19	X_1_X_2_Y	[31] ^b^
*Loxosceles laeta*	Araneomorphae, Haplogyne: Sicariidae	1 AD, 1 SAD	breeding	23	X_1_X_2_Y	this study
*Pholcus phalangioides*	Araneomorphae, Haplogynae: Pholcidae	2 SAD, 2 AD	Czech Republic, Prague	25	X_1_X_2_Y	[31]
*Tegenaria ferruginea*	Araneomorphae, Entelegynae: Agelenidae	3 AD	Czech Republic, Prague	40	neo-X_1_X_2_X_3_X_4_X_5_Y	[18] ^c^
		1 AD	Greece, Macedonia, 1 km west of Pentalofos	40	neo-X_1_X_2_X_3_X_4_X_5_Y	this study

^a^ AD, adult; SAD, subadult. Data at parentheses refer to specimens used for determination of basic karyotype data. ^b^ Referred as *Loxosceles spinulosa* in this study. ^c^ Referred as *Malthonica ferruginea* in this study.

## Supplementary Materials

The following are available online at https://www.mdpi.com/2073-4425/11/8/849/s1, Supplementary File 1: Figure S1. Comparative genomic hybridisation in analysed spiders—extended file with separated channels. First column: DAPI images (blue); Second column: hybridisation patterns generated by the male genomic probes (red); Third column: hybridisation patterns generated by the female genomic probes (green). Fourth column: merged images of both genomic probes. The numbering system of individual pictures or insets and the markings of sex chromosomes mirror those used in Figure 1, Figure 2, Figure 3 and Figure 4. (1b) *Linothele megatheloides*, (1e) *Grammostola* aff. *porteri*, (2b, 2c, 2e, 2f) *Poecilotheria formosa*, (2i) *Pterinochilus lugardi*, (3a-b) *Kukulcania* aff. *hibernalis*, (3c) *Loxosceles laeta*, (3d) *L. simillima*, (3e–f) *Pholcus phalangioides*, (4a–c) *Tegenaria ferruginea*, (4d) *Atrophothele socotrana*. Bar = 10 μm. Supplementary File 2: Figure S2. Male mitotic and meiotic data of analysed spiders. (a, b) *Grammostola* aff. *porteri*: (a) mitotic metaphase (2n = 72), most chromosomes biarmed. X_1_ chromosome is metacentric. Supposed X_2_ chromosome shows monoarmed morphology. Chromosomes of CSCPs belong to two pairs, metacentric (CSCP1) and submetacentric ones (CSCP2). These chromosomes form a cluster and display a precocious separation of sister chromatids except for a short arm of submetacentric pair; (b) metaphase I encompassing 35 bivalents and univalents X_1_ and X_2_, which are associated in the middle of the plate. Most bivalents include one chiasma only. (c, d) *Loxosceles laeta* (2n = 23): (c) mitotic metaphase comprising biarmed chromosomes (sc—supposed secondary constriction). Inset: submetacentric Y chromosome (ce—centromere); (d) metaphase I, note sex chromosome trivalent composed of two X chromosomes and Y microchromosome. Bivalents contain one or two chiasmata. (e, f) *Atrophothele socotrana*: (e) mitotic metaphase (2n = 68), most chromosomes moarmed. Majority of biarmed chromosomes belong to longest elements; (f) diplotene, most bivalents include one chiasma. Inset: trivalent from another diplotene; centromeres of monoarmed chromosomes are expressed as tiny dark regions (bi—heteromorphic bivalent, ce—centromere, t—sex chromosome trivalent, asterisk—chiasmata connecting chromosomes of trivalent). Bar = 10 µm except for insets of c (2 µm) and f (5 µm). Supplementary File 3: Figure S3. Male karyotypes of *Atrophothele socotrana* constructed from meiotic metaphase II. (a) haploid karyotype containing three X chromosomes (n = 35). While one X chromosome is metacentric, other two display monoarmed morphology; (b) haploid karyotype including metacentric Y chromosome (n = 33). Bar = 10 µm.
