# Supplementary material for: Patterns of Sex Chromosome Differentiation in Spiders: Insights from Comparative Genomic Hybridisation
Source: Genes (Basel). 2020 Jul 24;11(8):849. doi: 10.3390/genes11080849 (PMC7466014; doi:10.3390/genes11080849)
Supplement: Supplementary file 1 [file genes-11-00849-s001.zip › Supplementary_Files_revised/Supplementary_File_1_Figure_S1_merged.pdf]

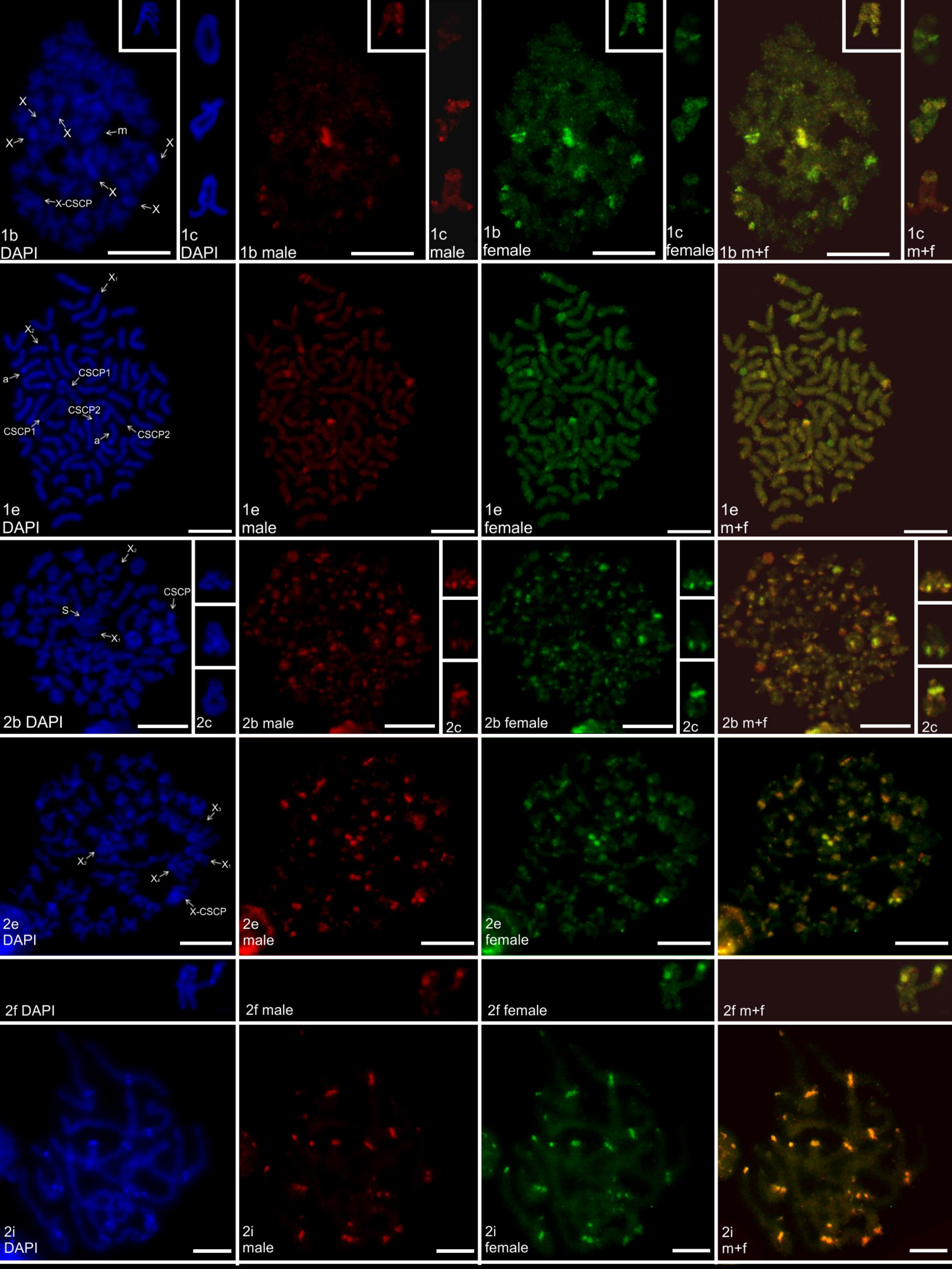
1b DAPI
X
X
m
X
X
X
X-CSCP
X
1c DAPI
1b male
1c male
1b female
1c female
1b m+f
1c m+f
1e DAPI
X1
X2
a
CSCP1
CSCP2
CSCP1
a
CSCP2
1e male
1e female
1e m+f
2b DAPI
X2
CSCP
S
X1
2c
2b male
2c
2b female
2c
2b m+f
2c
2e DAPI
X3
X2
X1
X4
X-CSCP
2e male
2e female
2f DAPI
2f male
2f female
2f m+f
2i DAPI
2i male
2i female
2i m+f

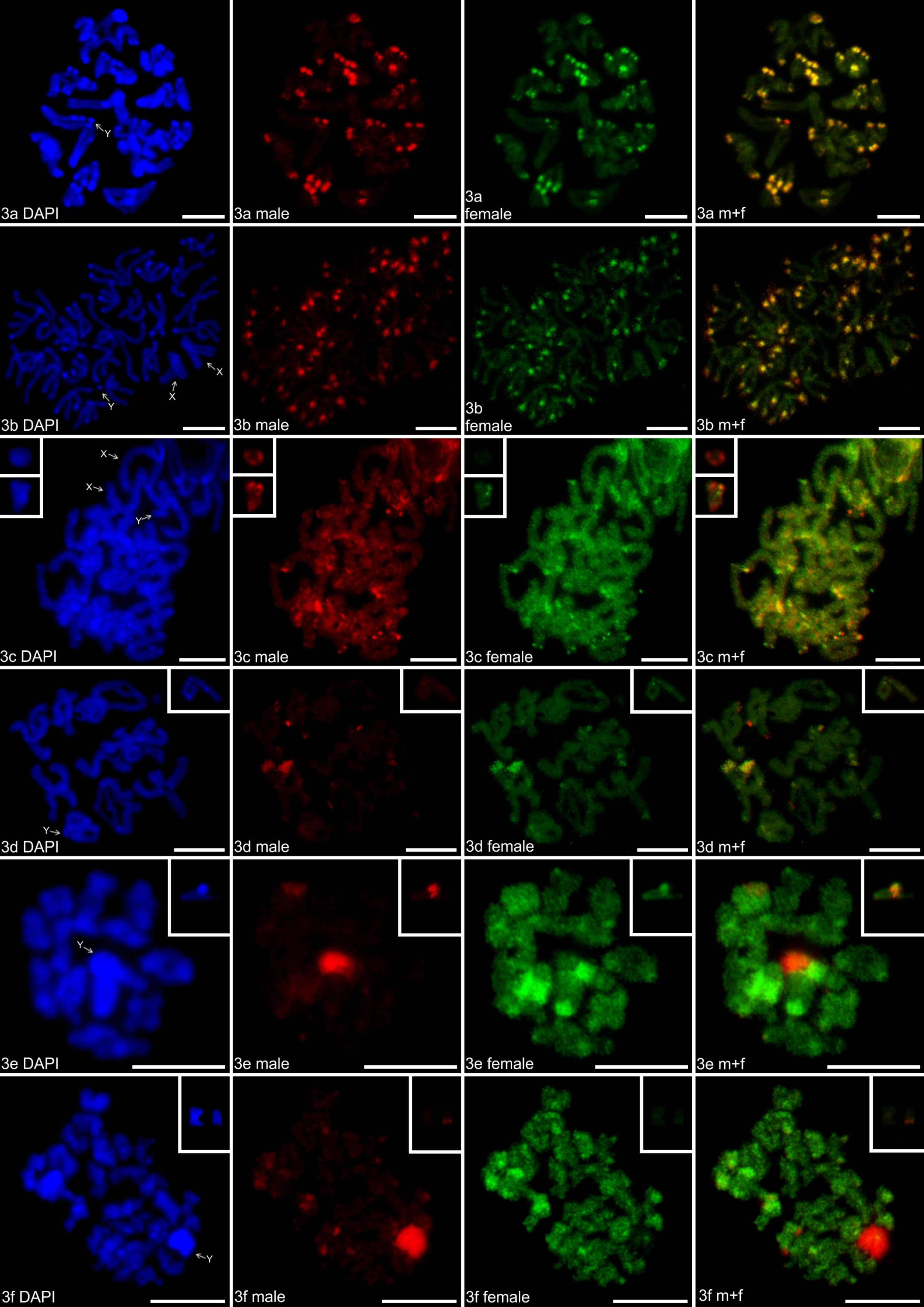

3a DAPI
Y
3a male
3a female
3a m+f
3b DAPI
X
X
Y
3b male
3b female
3b m+f
3c DAPI
X
X
Y
3c male
3c female
3c m+f
3d DAPI
Y
3d male
3d female
3d m+f
3e DAPI
Y
3e male
3e female
3e m+f
3f DAPI
Y
3f male
3f female
3f m+f

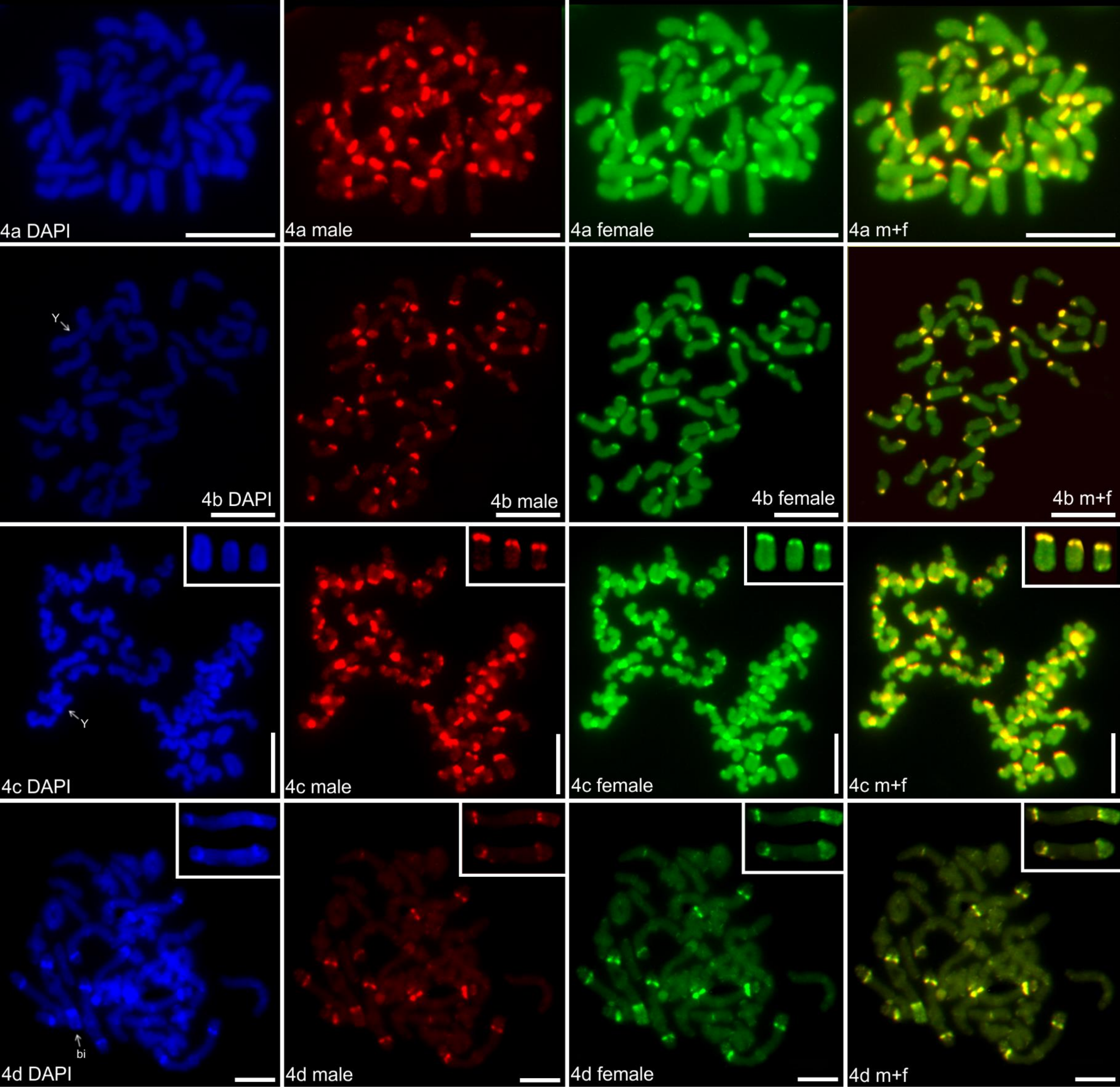

Supplementary File 1: Figure S1. Comparative genomic hybridization in analysed spiders – extended file with separated channels. First column: DAPI images (blue); Second column: hybridization patterns generated by the male genomic probes (red); Third column: hybridization patterns generated by the female genomic probes (green). Fourth column: merged images of both genomic probes. The numbering system of individual pictures or insets and the markings of sex chromosomes mirror those used in Figs. 1–4. Bar = 10 μm.
