# Supplementary figures and images for: Patterns of Sex Chromosome Differentiation in Spiders: Insights from Comparative Genomic Hybridisation
Source: Genes (Basel). 2020 Jul 24;11(8):849. doi: 10.3390/genes11080849 (PMC7466014; doi:10.3390/genes11080849)

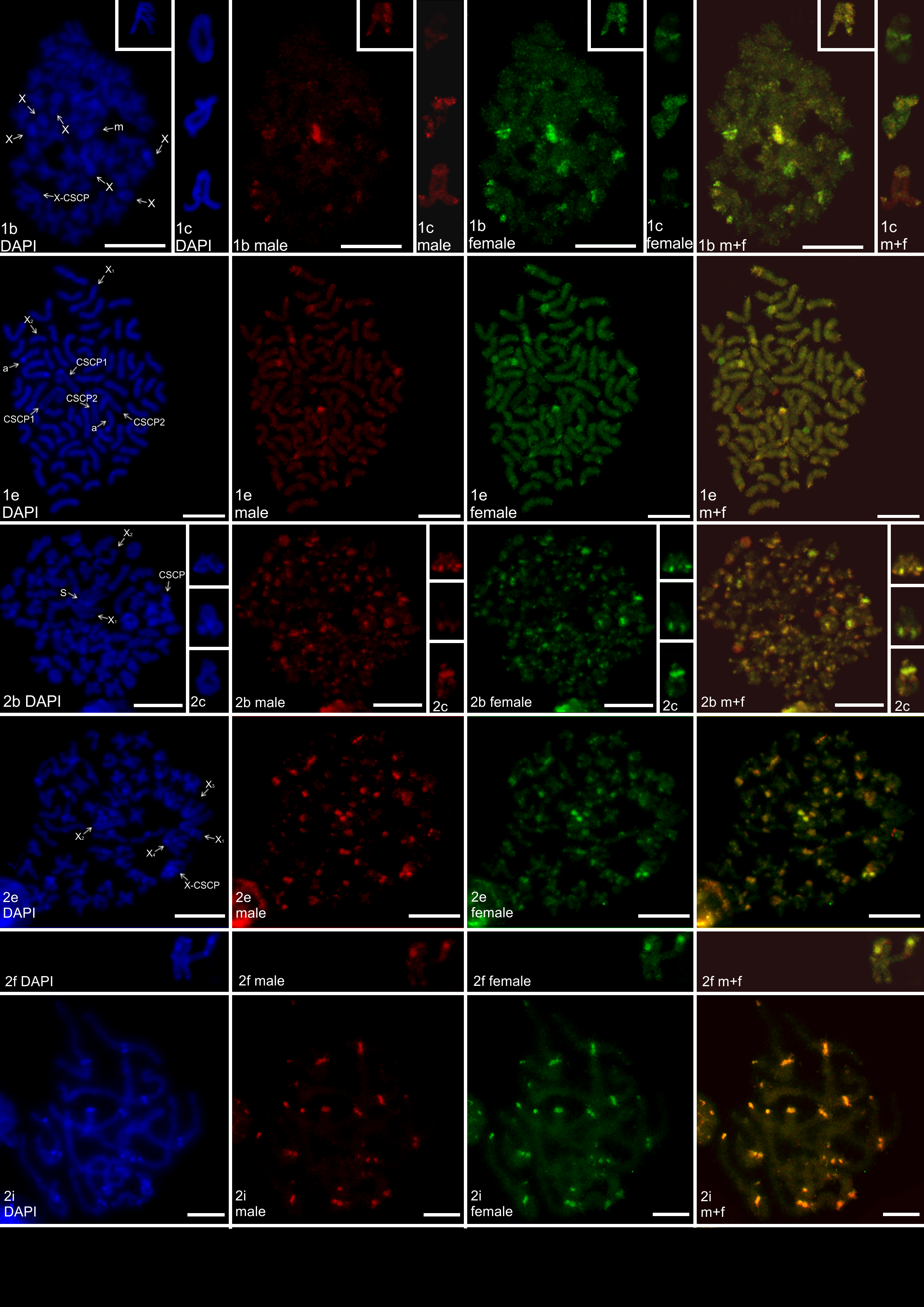

Supplement: Supplementary file 1 [file genes-11-00849-s001.zip › Supplementary_Files_revised/Supplementary_File_1_Figure_S1a.tif]

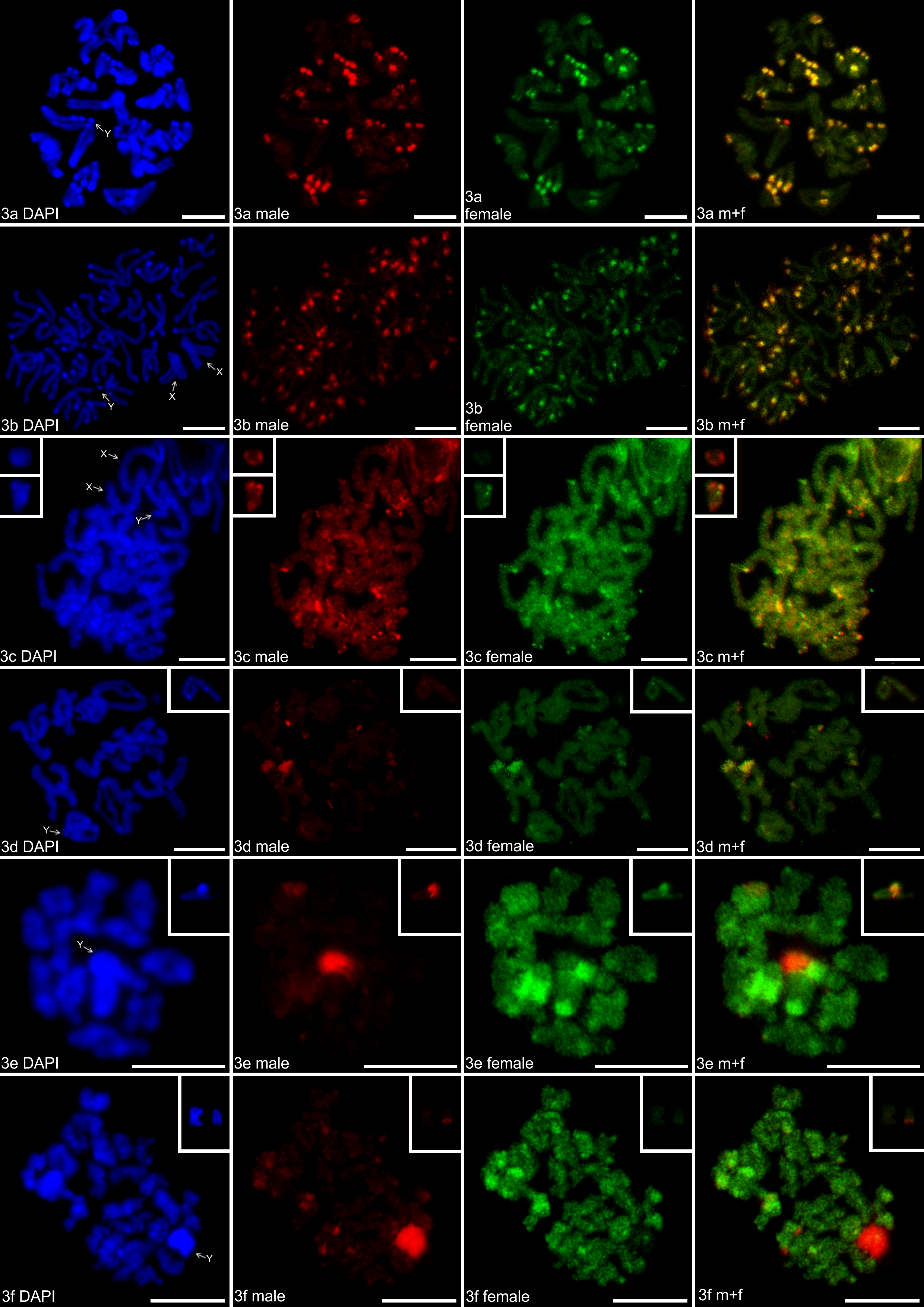

Supplement: Supplementary file 1 [file genes-11-00849-s001.zip › Supplementary_Files_revised/Supplementary_File_1_Figure_S1b.TIF]

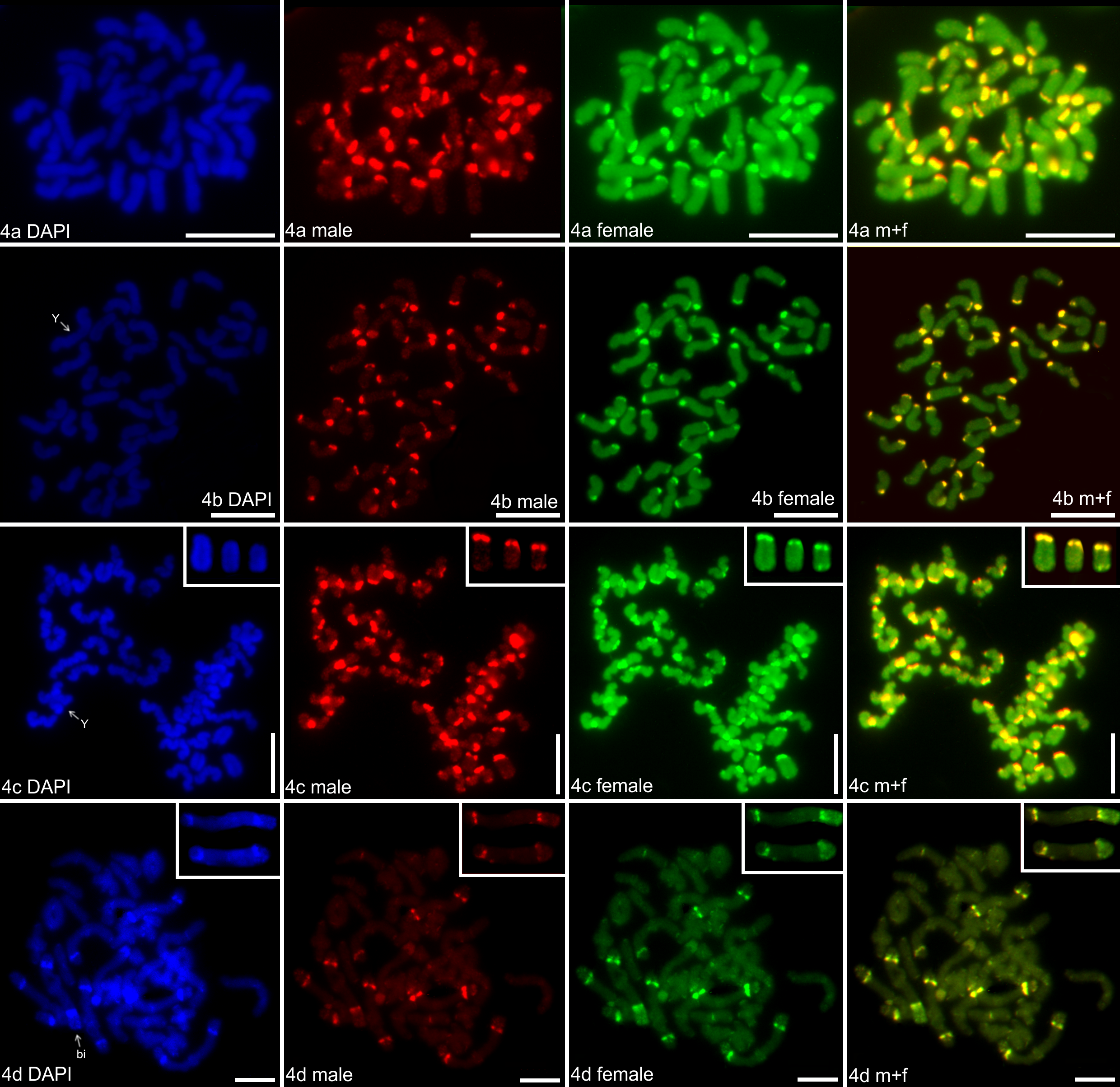

Supplement: Supplementary file 1 [file genes-11-00849-s001.zip › Supplementary_Files_revised/Supplementary_File_1_Figure_S1c.TIF]

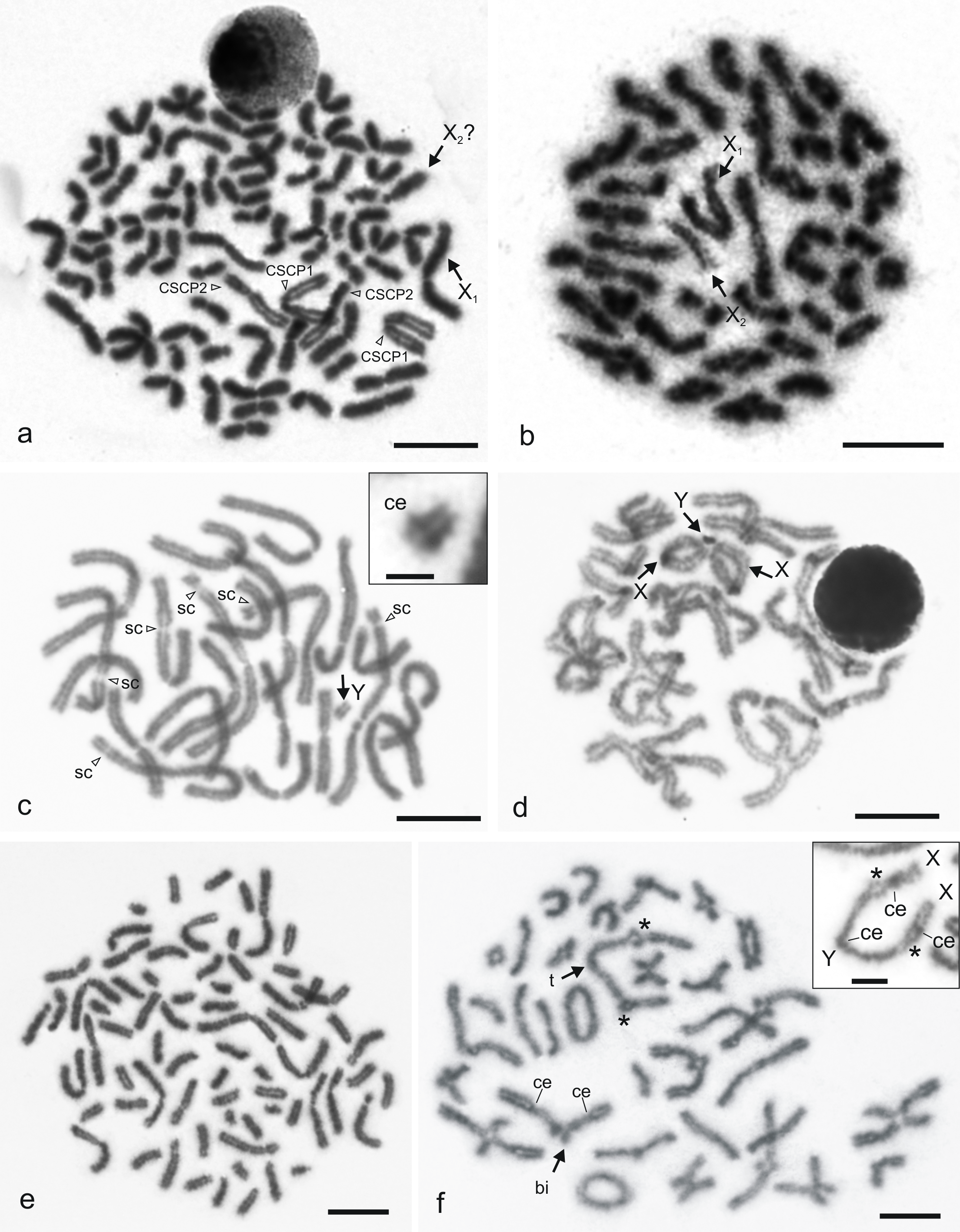

Supplement: Supplementary file 1 [file genes-11-00849-s001.zip › Supplementary_Files_revised/Supplementary_File_2_Figure_S2.tif]

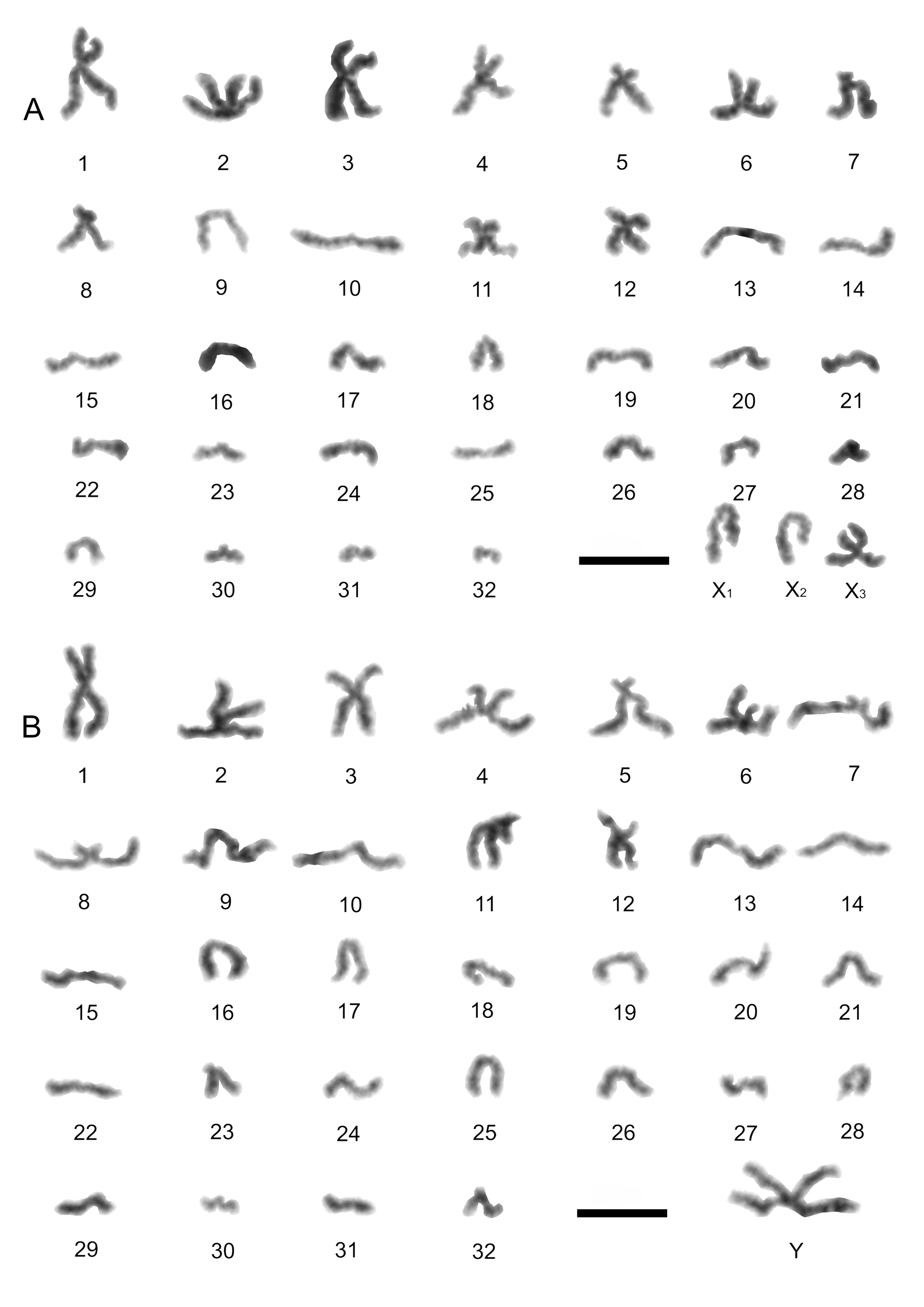

Supplement: Supplementary file 1 [file genes-11-00849-s001.zip › Supplementary_Files_revised/Supplementary_File_3_Figure_S3.TIF]
